# Organic selenium supplement partially alleviated diquat-induced oxidative insults and hepatic metabolic stress in nursery pigs

**DOI:** 10.1017/S0007114520000689

**Published:** 2020-07-14

**Authors:** Nicole Doan, Yanhong Liu, Xia Xiong, Kwangwook Kim, Zhaohai Wu, David M. Bravo, Alexandra Blanchard, Peng Ji

**Affiliations:** 1 Department of Nutrition, University of California, Davis, CA 95616, USA; 2 Department of Animal Science, University of California, Davis, CA 95616, USA; 3 Key Laboratory of Agro-ecological Processes in Subtropical Region, National Engineering Laboratory for Pollution Control and Waste Utilization in Livestock and Poultry Production, Institute of Subtropical Agriculture, Chinese Academy of Sciences, Changsha, Hunan 210125, Peopleʼs Republic of China; 4 Institute of Animal Sciences, Chinese Academy of Agricultural Sciences, Beijing 100193, Peopleʼs Republic of China; 5 Pancosma SA, 1180 Rolle, Switzerland

**Keywords:** Selenium sources, Diquat challenge, Oxidative stress, Nursery pigs, Hepatic metabolomics

## Abstract

The study investigated antioxidant effects of Se on resilience to diquat-induced oxidative stress in nursery pigs. Thirty-five weaned pigs were individually housed and randomly assigned to one of the five treatments. Pigs were (1) fed a basal diet and intraperitoneally injected with sterile saline (negative control), (2) fed the basal diet and injected with diquat solution (positive control, PC), or fed the basal diet supplemented with 0·3 mg Se/kg as (3) sodium selenite (SS), (4) soyabean protein-chelated Se (SC) or (5) selenised yeast (SY) and intraperitoneally injected with diquat. Pigs were fed the experimental diets for 17 d and injected with diquat at 10 mg/kg body weight or saline on the 11th day of the study (day 0 post-injection (PI)). Diquat exposure induced acute stress and innate immune activation (*P* < 0·05) at 6 h PI and compromised (*P* < 0·05) plasma glutathione peroxidase activity on day 2 PI, which was accompanied by an increase in plasma malondialdehyde at 6 h and day 2 PI (*P* < 0·10). Organic Se, particularly SY, enhanced (*P* < 0·05) endogenous antioxidant activity in various aspects compared with the PC group. The growth rate and feed intake from day 0 to day 7 PI were significantly lower in the PC, SS and SC groups than the NC group (*P* < 0·05). Untargeted metabolomics analysis revealed that twenty-two hepatic metabolites (false discovery rate < 0·15) associated with lipid and cellular antioxidant metabolism were altered by diquat. SY restored hepatic metabolic profiles in some but not all samples.

Weaning is a stressful event that jeopardises the health and growth of piglets in the swine industry. Heightened oxidative biomarkers have been detected in the gut epithelium, liver and blood circulation of weanling piglets^(1,2)^, suggesting the presence of oxidative stress. Improper management, environmental stressors (i.e. heat stress), oxidised feed, early weaning and infections have all been reported to induce or exacerbate oxidative insults in weaned piglets^(1,3,4)^. Poor post-weaning growth rate has been shown to be associated with heightened blood indicators of oxidative stress and compromised antioxidants pre- and post-weaning^(1)^.

Dietary strategies that enhance endogenous antioxidant capacity are a promising approach to mitigate oxidative stress in weanling pigs^(5–7)^. Se is the cofactor for a number of selenoproteins (i.e. glutathione peroxidises (GPx), thioredoxin reductases, selenoprotein-P) that are critically involved in antioxidative, anti-inflammatory and pro-survival processes^(8,9)^. A good body of evidence suggests that the bioavailability of Se and its effects on antioxidant capacities in humans and animals are influenced by its chemical forms^(10–12)^. For example, in weaned pigs, dietary supplementation of dl-selenomethionine or Se-enriched probiotics (organic Se sources) enhanced antioxidant measurements to a greater extent compared with sodium selenite (SS)^(13,14)^. In comparison with SS and selenised yeast (SY), organic 2-hydroxy-4-methylselenobutanoic acid further increased Se concentration in plasma, liver and muscle of growing gilts^(15)^. Moreover, with increasing dose of Se supplement (5–20 mg Se/kg diet), Se-enriched yeast (organic Se) was associated with more rapid increase in tissue Se deposition compared with SS (inorganic Se)^(16)^. However, supplementing Se above 5 mg/kg adversely affected intake and growth, of which a sharper decline was observed with inorganic Se compared with organic Se^(16)^.

All studies discussed above were conducted in healthy pigs under physiological conditions. How Se of different sources affects antioxidant capacity and growth of weanling pigs under oxidative insults is largely unknown. A standardised oxidative stress model is thus critical. Diquat is a bipyridyl herbicide that could utilise molecular O_2_ to generate superoxide anion radical^(17)^. Exposure to diquat caused rapid production of reactive oxygen species and tissue oxidative damage in laboratory animals^(18,19)^ and livestock species, including pigs^(7,20–22)^. The liver has been shown to be the main target organ of diquat-induced oxidative insults^(18,19)^. The present study evaluated effects of Se supplements on resilience and hepatic metabolic response to diquat-induced oxidative stress in weanling pigs. Three sources of Se supplements were compared in the present study including SS, SY and soya-chelated Se (SC), which is a novel Se supplement that was produced by chelating SS with hydrolysed soyabean protein.

## Materials and methods

### Animal care and experimental design

The protocol (no. 19322) for this experiment was approved by the Institute of Animal Care and Use Committee at the University of California, Davis. The experiment was conducted in Cole facility at University of California, Davis. A total of thirty-five cross-bred pigs (Duroc × Yorkshire × Landrace; initial body weight (BW) 9·72 (sd 1·39) kg) including twenty barrows and fifteen gilts were blocked by sex and litter and were randomly assigned to one of the five treatments (*n* 7/treatment) at 1 week after weaning (approximately 28 d of age). Pigs were individually housed in crates enriched with toys (pen size 0·61 × 1·22 m) with free access to experimental diet and water throughout the 17-d study.

Pigs in the negative control (NC) or positive control (PC) groups were fed a basal diet without Se supplement and intraperitoneally injected with sterile saline (NC) or diquat (PC) on the 11th day of the study (day 0 relative to injection). Pigs in Se supplement groups were fed the basal diet supplemented with 0·3 mg Se/kg in the form of SS, SC or SY and intraperitoneally injected with diquat on the 11th day of the study. The study lasted for 17 d covering 10 d pre- and 7 d post-injection (PI) of diquat. All Se products were provided by Pancosma, S.A. The Se content was 46 % (w/w) in SS, 2·9 % in SC and 0·22 % in SY. The basal diet was formulated to meet the nutrient requirements for nursery pigs with the exception of dietary Se concentration (<0·2 mg/kg as-fed basis) which was below the Se recommendation of 0·3 mg/kg (Table 1). Representative samples of experimental diets were submitted to Cumberland Valley Analytical Services for analyses of macronutrients and University of Missouri Agricultural Experiment Station Chemical Laboratories for analysis of Se content. Pigs were weighed at the beginning of the study (day –10), the day of diquat injection (day 0) and at the end of the experiment (day 7 PI). Daily feed allotments were recorded to calculate average daily gain, average daily feed intake and gain:feed ratio. Pigs were euthanised on day 7 PI for tissue sample collection.

**Table 1.** Ingredient and nutrient composition of the basal diets

Items	Basal diet*
Ingredients (%)	
Maize	44·51
Dried whey	15·00
Soyabean meal	14·00
Fishmeal	10·00
Soya protein concentrate	7·00
Lactose	6·00
Soyabean oil	2·00
Limestone	0·56
l-Lysine·HCl	0·15
dl-Methionine	0·06
l-Threonine	0·02
Salt	0·40
Vitamin-mineral premix† ‡	0·30
Total	100·00
Analysed nutrients (% as-fed)	
DM	89·5
Crude protein	21·21
Acid-detergent fibre	3·49
Neutral-detergent fibre	7·61
Ca	0·89
P	0·59
Se§ (mg/kg)	<0·20

* Pigs in the negative control and positive control groups were fed the basal diet without Se supplements. Pigs in Se-supplement groups were fed the basal diet supplemented with 0·3 mg Se/kg diet from sodium selenite (SS), soyabean protein-chelated Se (SC) or selenised yeast (SY).

† No Se supplement was added to the basal diet. Se supplement was first mixed well with the premix and 500 g of maize meal before mixing with other ingredients of the basal diet. Se accounts for 46, 2·9 and 0·22 % in SS, SC and SY, respectively. SS, SC and SY were supplemented at 0·66, 10·34 and 136·36 mg/kg in the basal diet, respectively, to provide 0·3 mg Se/kg diet.

‡ Provided the following quantities of vitamins and micro-minerals per kg of complete diet: Vitamin A as retinyl acetate, 3340·8 μg; vitamin D_3_ as cholecalciferol, 55·2 μg; vitamin E as dl-*α* tocopheryl acetate, 44·2 μg; vitamin K as menadione dimethylpyrimidinol bisulphite, 1·42 mg; thiamin as thiamine mononitrate, 0·24 mg; riboflavin, 6·59 mg; pyridoxine as pyridoxine hydrochloride, 0·24 mg; vitamin B_12_, 0·03 mg; d-pantothenic acid as d-calcium pantothenate, 23·5 mg; niacin, 44·1 mg; folic acid, 1·59 mg; biotin, 0·44 mg; Cu, 20 mg as copper sulphate and copper chloride; Fe, 126 mg as ferrous sulphate; iodine, 1·26 mg as ethylenediamine dihydriodide; Mn, 60·2 mg as manganese sulphate; and Zn, 125·1 mg as zinc sulphate.

§ Analysed Se content of SS, SC and SY diets was 0·4 mg/kg.

### Diquat injection

On day 0 PI, all pigs except those in the NC group were intraperitoneally injected with diquat (Diquat dibromide monohydrate; Sigma-Aldrich, Inc.) at 10 mg diquat ion/kg BW. Diquat injections were prepared by dissolving diquat powder in 0·9 % sterile saline solution to a final concentration of 10 mg/ml. Pigs in the NC group were intraperitoneally injected with 10 ml of 0·9 % sterile saline solution.

### Total and differential blood cell counts

Whole blood samples were collected from jugular vein at 0 h (prior to diquat injection), 6 h, 24 h, 2 d, 4 d and 7 d PI and submitted to Comparative Pathology Laboratory at University of California, Davis. Total and differential blood cell counts were determined using a multiparameter, automated haematology analyser (Drew/ERBA Scientific 950 FS Hematological Analyzer; Drew Scientific Inc.) following a protocol that was previously optimised for porcine blood samples.

### Plasma antioxidant enzymes, lipid peroxidation and cortisol concentration

Plasma was harvested from a portion of blood samples through centrifugation at 1500 ***g*** for 15 min at 4°C and stored at –80°C until analysis. The activities of GPx (catalogue no. 703102), superoxide dismutase (SOD; catalogue no. 706002), total antioxidant capacity (TAC; catalogue no. 709001) and plasma malondialdehyde (MDA; catalogue no. 700870) concentration were analysed through colorimetric assays using commercial kits (Cayman Chemical). The result of SOD activity was presented as U/ml with the unit of SOD defined as the amount of enzyme needed to exhibit 50 % dismutation of the superoxide radical. The activity of GPx was indirectly detected through determining the oxidation of NADPH to NADP^+^ and presented as nmol/min per ml. The TAC was calculated based on a standard curve generated using Trolox and presented as mmol Trolox/ml. The total concentration of plasma MDA (µmol/ml) was measured using a thiobarbituric acid-reactive substances assay.

#### Plasma cortisol concentration

The concentration of cortisol in plasma samples was measured using a commercial ELISA kit (catalogue no. KGE008B; R&D Systems, Inc.). The result was calculated based on the absorbance at 450 nm and referenced to a standard curve.

### Hepatic antioxidant enzymes and lipid peroxidation

Liver samples collected after euthanasia were snap-frozen in liquid N_2_ and stored at –80°C until analysis. Liver tissue (approximately 200 mg) was homogenised using a glass tissue grinder in 2 ml ice-cold lysis buffer containing a cocktail of protease inhibitors (Sigma-Aldrich, Inc.). The liver homogenate was centrifuged at 10 000 ***g*** for 5 min at 4°C to harvest supernatant for assays of antioxidant enzymes and lipid peroxidation. The activities of GPx, SOD, TAC and catalase, and MDA concentration in liver samples were analysed using the same kits stated previously. The activity of catalase in liver samples was determined using a commercial kit (catalogue no. 707002; Cayman Chemical). The kit measures product formation from the reaction of catalase with methanol in the presence of hydrogen peroxide (H_2_O_2_). The concentration of total protein in liver homogenate was determined using Pierce Micro BCA Protein Assay kit (Thermo Scientific). The final results of antioxidant parameters were normalised with total protein concentration in each sample. The results of GPx, SOD, TAC and catalase were expressed in nmol/min per mg protein, U/mg protein, mmol Trolox/mg protein, μmol formaldehyde/min per mg protein, respectively. The MDA concentration was expressed in µmol/mg protein.

### Untargeted metabolomics analysis

Liver samples from the NC, PC and SY groups were submitted for metabolomics analysis. The analysis was performed by the NIH West Coast Metabolomics Center using GC (Agilent 6890) coupled with time-of-flight MS (Leco Pegasus IV). Samples were processed based on the modified procedures as previously stated by others^(23,24)^. Briefly, liver samples (approximately 10 mg) were mixed with 1 ml extraction solvent (acetonitrile, isopropanol and water in proportion 3:3:2) and homogenised for 45 s. Then, the homogenate was centrifuged for 2 min at 14 000 ***g*** to generate supernatant, which was completely dried using a cold-trap vacuum concentrator (Labconco Centrivap). Dried samples were subsequently resuspended in 500 μl of a 50 % aqueous acetonitrile solution and centrifuged (14 000 ***g*** for 2 min) to remove membrane lipids and TAG. The sample was concentrated and derivatised and then mixed with 1 μl of internal markers (C8–C30 fatty acid methyl esters). The derivatised sample was injected for GC time-of-flight MS analysis as previously described^(24)^. All samples were analysed in one batch. The raw spectral data were first processed using Leco ChromaTOF software (version 2.32) for peak identification and mass spectra deconvolution. The spectral data were further refined using the BinBase algorithm^(25)^. All metabolite spectra in BinBase were matched against the Fiehn mass spectral library and the NIST spectral library based on the retention index and mass spectrum. The mapped metabolites were reported for compound names and signal intensity of peak height (counts per spectrum).

### Statistical and bioinformatics analysis

Data were verified for normal distribution using the UNIVARIATE procedure of SAS and analysed using the PROC MIXED of SAS (SAS Institute). The statistical model included treatment as the fixed effect, while blocks and pigs were included as random effects. Least squares means of treatment effect were calculated, and the mean separation was performed through PDIFF function when significant treatment effect was detected. Statistical significance was considered at *P* ≤ 0·05, and trend towards significance was considered at *P* ≤ 0·10.

Statistical and bioinformatics analyses for identified and unidentified metabolites were performed separately. The metabolomics data of peak intensity were subjected to natural-logarithmic transformation and autoscaling before a Studentʼs *t* test that was performed using PROC MULTTEST of SAS (version 9.4) with Benjamini–Hochberg adjustment for multiple tests. Significance was declared at *P* ≤ 0·05, and the false discovery rate-adjusted *P* value (*q* value) ≤ 0·15. Metabolites that were significantly impacted were uploaded to MetaboAnalyst (https://www.metaboanalyst.ca/) for pathway enrichment analysis and clustering analysis. To identify the predictors of metabolic difference, multivariate modelling including principal component analysis (PCA) was performed on the data set of identified metabolites through built-in R statistics in MetaboAnalyst. Score plots were generated to visualise the top two sources of variance.

## Results

### Growth and feed intake

**Fig. 1.**
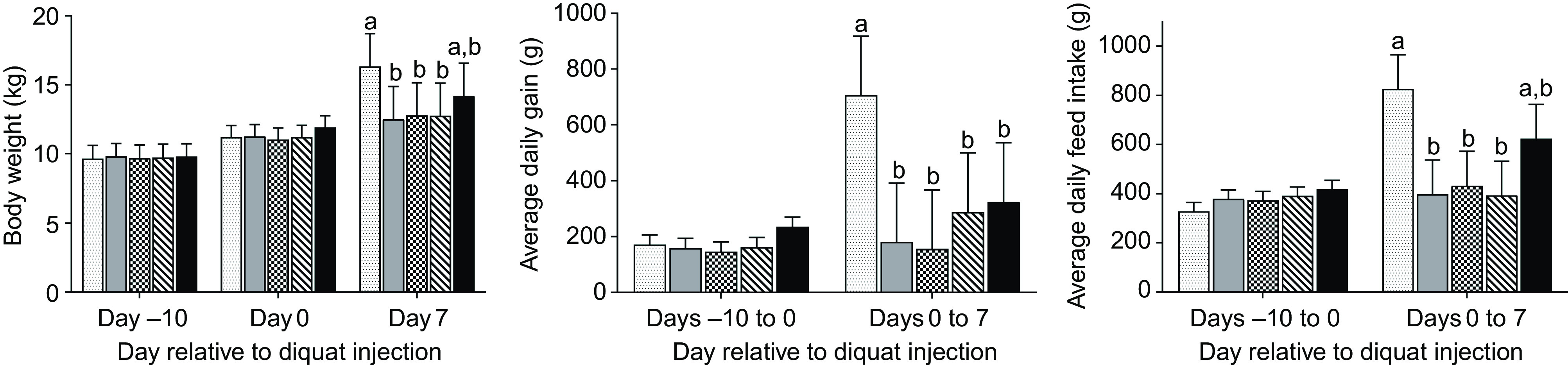
Growth performance and feed intake of piglets (*n* 7/treatment) in response to diquat challenge and dietary selenium supplementation. Values were presented as least square means with their standard errors. ^a,b^ Mean values with unlike letters were significantly different (*P* < 0·05). 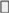, Negative control without diquat challenge nor dietary selenium supplementation; 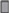, positive control with diquat challenge without dietary selenium supplementation; 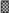, sodium selenite group with diquat challenge; 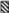, soya protein-chelated selenium group with diquat challenge; 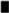, selenised yeast group with diquat challenge.

No difference was observed in BW among treatment groups on day –10 or day 0 (Fig. 1). The final BW of pigs in the NC group was significantly higher (*P* < 0·05) than that of diquat-challenged pigs on day 7 PI, with the exception that the final BW of pigs in SY was not statistically different from any other group. Neither average daily gain nor average daily feed intake was different among groups from day –10 to day 0. In comparison with the NC group, diquat injection significantly reduced (*P* < 0·05) average daily feed intake and average daily gain from day 0 to day 7 PI with the exception that the average daily feed intake of SY was not statistically different from the other groups.

### Leucocyte profiles

Total leucocyte count did not differ among treatments prior to diquat injection (Table 2). The cell counts of leucocyte and neutrophil were significantly greater in the PC group than other groups at 6 h PI (*P* < 0·05). Lymphocyte count was significantly greater in Se-supplemented groups than that of the NC group at 6 h PI (*P* < 0·05). All groups injected with diquat had a higher ratio of neutrophils:lymphocytes than the NC group at 6 h PI (*P* < 0·05). Pigs in the PC and SS groups had more leucocytes than the NC group and more lymphocytes than the NC and SY groups on day 4 PI (*P* < 0·05).

**Table 2.** Effect of dietary supplementation of selenium on leucocyte profiles and plasma cortisol concentration of weanling pigs in response to diquat injection (*n* 6) (Mean values with their standard errors; 6–7 observations per least square mean value)

Items	Diquat challenge					sem	*P*
	NC	PC	SS	SC	SY		
Day 0 before infection							
Leucocytes (10^3^/μl)	11·61	11·45	12·24	11·11	10·50	1·78	0·97
Neutrophils (10^3^/μl)	5·04	5·29	5·20	5·46	4·61	1·18	0·99
Lymphocytes (10^3^/μl)	5·37	4·80	5·60	4·35	4·62	0·98	0·83
Neutrophils:lymphocytes	0·925	1·098	1·007	1·342	1·120	0·359	0·88
Cortisol (ng/ml)	26·28	29·18	27·76	28·26	29·51	3·82	0·86
6 h PI							
Leucocytes (10^3^/μl)	13·96^b^	17·54^a^	12·49^b^	12·86^b^	12·36^b^	1·78	<0·05
Neutrophils (10^3^/μl)	6·67^b^	11·42^a^	7·77^b^	8·34^b^	7·78^b^	1·18	<0·05
Lymphocytes (10^3^/μl)	5·88^a^	4·58^a,b^	3·78^b^	3·29^b^	3·43^b^	0·98	<0·05
Neutrophils:lymphocytes	1·428^b^	3·089^a^	2·393^a^	3·063^a^	3·125^a^	0·359	<0·01
Cortisol (ng/ml)	26·70^c^	88·55^a^	63·72^b^	56·01^b^	47·26^b,c^	11·01	<0·01
24 h PI							
Leucocytes (10^3^/μl)	14·12	16·24	14·81	13·75	13·89	1·78	0·82
Neutrophils (10^3^/μl)	5·89	7·99	7·53	6·57	6·53	1·18	0·62
Lymphocytes (10^3^/μl)	6·95	6·77	6·01	5·76	5·90	0·98	0·82
Neutrophils:lymphocytes	0·932	1·235	1·391	1·278	1·248	0·359	0·86
Cortisol (ng/ml)	29·62	48·05	32·55	39·00	32·56	4·95	0·09
2 d PI							
Leucocytes (10^3^/μl)	14·94	17·95	16·26	17·05	17·29	1·82	0·73
Neutrophils (10^3^/μl)	7·09	8·88	8·25	9·13	9·51	1·21	0·52
Lymphocytes (10^3^/μl)	6·43	7·68	6·75	6·53	6·32	1·00	0·79
Neutrophils:lymphocytes	1·151	1·232	1·271	1·507	2·169	0·365	0·09
Cortisol (ng/ml)	26·31	38·27	29·10	34·67	28·65	4·70	0·07
4 d PI							
Leucocytes (10^3^/μl)	14·71^b^	18·49^a^	18·97^a^	16·56^a,b^	15·31^a,b^	1·86	<0·05
Neutrophils (10^3^/μl)	6·96	8·44	9·62	8·21	8·29	1·23	0·53
Lymphocytes (10^3^/μl)	6·68^b^	8·89^a^	8·05^a^	7·10^a,b^	5·89^b^	1·02	<0·05
Neutrophils:lymphocytes	1·270	0·966	1·206	1·325	1·507	0·372	0·82
Cortisol (ng/ml)	22·24	28·22	22·26	38·28	21·15	5·25	0·07
7 d PI							
Leucocytes (10^3^/μl)	11·28	13·84	12·63	11·79	11·31	1·85	0·81
Neutrophils (10^3^/μl)	4·87	7·01	5·65	5·68	5·22	1·23	0·67
Lymphocytes (10^3^/μl)	5·32	5·59	5·74	5·09	4·97	1·02	0·98
Neutrophils:lymphocytes	0·969	1·362	1·073	1·337	1·159	0·371	0·87
Cortisol (ng/ml)	27·68	30·33	28·25	33·47	22·01	5·56	0·39

NC, negative control; PC, positive control; SS, sodium selenite; SC, soyabean protein-chelated Se; SY, selenised yeast; PI, post-injection.

^a,b,c^ Mean values in a row with unlike superscript letters were significantly different (*P* < 0·05).

### Plasma cortisol concentration

Treatment did not affect plasma cortisol concentration on day 0 PI (Table 2). Diquat injection significantly increased (*P* < 0·01) plasma cortisol at 6 h PI compared with the NC group. In comparison with the PC group, dietary supplementation of Se significantly reduced plasma cortisol at 6 h (*P* < 0·01) with the greatest reduction induced by SY. There was a trend of treatment effect at 24 h (*P* = 0·09), on day 2 (*P* = 0·07) and day 4 PI (*P* = 0·07). Plasma cortisol was the highest in the PC group at 24 h and on day 2 and the lowest in the SY group on day 2 and day 4.

### Lipid peroxidation and antioxidant activity

Supplementation of SY enhanced plasma GPx activity compared with NC, PC and SS groups on day 0 PI (*P* < 0·05; Table 3). The plasma GPx activity was lower in the PC and SS groups compared with the SY group at 6 h PI or compared with both the SY and NC groups on day 2 PI (*P* < 0·05). Despite lack of treatment effect on day 4 PI, a trend of treatment effect on plasma GPx activity was detected on day 7 PI (*P* = 0·09), wherein highest activity was observed in the SY group, followed by NC, SS, SC and PC. There was a significant treatment effect on plasma SOD activity at 24 h, on days 2, 4 and 7 PI (*P* < 0·05). At 24 h, the plasma SOD activity was higher in SC and SY compared with NC and PC (*P* < 0·05) and was higher in SS compared with NC. SY and SS had the highest plasma SOD activity on day 2 and day 4 PI. It was significantly different in comparison with NC on day 2 PI or both NC and PC on day 4 PI (*P* < 0·05). On day 7 PI, the highest plasma SOD activity was detected in SC, which was significantly different from SS, NC and PC (*P* < 0·05). Pigs in the SY group had higher SOD activity than those in the PC group on day 7 PI (*P* < 0·05). Plasma TAC was significantly higher in the SS and SC groups than in the NC and PC groups on day 2 PI (*P* < 0·05). There was a trend of treatment effect on plasma TAC on day 0, at 6 h, and 24 h PI (*P* ≤ 0·10). It was generally greater in the Se-supplemented groups compared with the PC and NC groups. There was a trend (*P* = 0·09) of treatment effect on plasma MDA concentration at 6 h and on day 2 PI. Highest plasma concentration of MDA was observed in PC in both time points.

**Table 3.** Effect of dietary supplementation of selenium on plasma antioxidant parameters of weanling pigs in response to diquat challenge (*n* 6) (Mean values with their standard errors)

Items	Diquat challenge					sem	*P*
	NC	PC	SS	SC	SY		
0 d (baseline)							
SOD (U/ml)	29·69	31·01	32·29	31·37	36·61	3·29	0·51
GPx (nmol/min per ml)	303·1^b^	299·1^b^	301·9^b^	391·4^a,b^	421·1^a^	46·37	<0·05
TAC (mmol Trolox/ml)	1·17	1·39	1·74	1·51	1·44	0·20	0·07
MDA (µmol/ml)	6·89	7·61	6·73	6·45	4·86	1·26	0·43
6 h PI							
SOD (U/ml)	31·87	31·11	41·50	37·56	35·24	4·51	0·25
GPx (nmol/min per ml)	350·6^a,b^	309·9^b^	320·1^b^	361·7^a,b^	427·0^a^	36·90	<0·05
TAC (mmol Trolox/ml)	1·30	1·31	1·76	1·47	1·49	0·18	0·08
MDA (µmol/ml)	5·87	8·55	6·26	4·98	6·36	1·07	0·09
24 h PI							
SOD (U/ml)	24·93^c^	30·76^b,c^	36·33^a,b^	42·20^a^	39·17^a^	2·94	<0·01
GPx (nmol/min per ml)	417·7	305·6	372·3	368·0	398·6	44·32	0·47
TAC (mmol Trolox/ml)	1·30	1·55	1·75	1·75	1·68	0·21	0·10
MDA (µmol/ml)	5·54	7·47	6·10	5·51	5·20	1·01	0·13
2 d PI							
SOD (U/ml)	28·73^b^	34·34^a,b^	39·17^a^	33·16^a,b^	38·55^a^	3·19	<0·05
GPx (nmol/min per ml)	397·3^a^	284·2^b^	306·8^b^	360·0^a,b^	394·8^a^	32·74	<0·05
TAC (mmol Trolox/ml)	1·34^b^	1·30^b^	1·66^a^	1·68^a^	1·46^a,b^	0·14	<0·05
MDA (µmol/ml)	4·81	7·46	5·52	6·78	5·38	0·82	0·09
4 d PI							
SOD (U/ml)	24·58^b^	28·95^b^	41·45^a^	34·77^a,b^	37·60^a^	2·64	<0·01
GPx (nmol/min per ml)	398·2	307·0	367·9	335·2	401·6	56·71	0·50
TAC (mmol Trolox/ml)	1·36	1·23	1·51	1·42	1·31	0·24	0·83
MDA (µmol/ml)	4·19	5·56	4·85	4·68	4·53	0·75	0·78
7 d PI							
SOD (U/ml)	27·70^b,c^	27·03^c^	30·35^b,c^	37·66^a^	33·57^a,b^	2·63	<0·05
GPx (nmol/min per ml)	376·1	222·4	358·9	306·1	403·3	48·08	0·09
TAC (mmol Trolox/ml)	1·41	1·40	1·59	1·31	1·37	0·15	0·70
MDA (µmol/ml)	4·17	4·67	5·25	4·88	4·93	0·99	0·95

NC, negative control; PC, positive control; SS, sodium selenite; SC, soyabean protein-chelated Se; SY, selenised yeast; baseline, before injection; SOD, superoxide dismutase; GPx, glutathione peroxidase; TAC, total antioxidant capacity; MDA, malondialdehyde; PI, post-injection.

^a,b,c^ Mean values within a row with unlike superscript letters were significantly different (*P* < 0·05).

Treatment significantly affected liver SOD and GPx activity (*P* < 0·05) and tended to affect liver catalase activity (*P* = 0·07) and MDA concentration (*P* = 0·09), but had no effect on liver TAC. The liver SOD activity was lower in the PC group than that in the NC and SC groups (*P* < 0·05; Table 4), whereas the liver GPx activity was the lowest in the SS group followed by the PC and SC groups and was the highest in the NC and SY groups (*P* < 0·05). Liver catalase activity and MDA concentration were the highest in the PC group compared with other groups.

**Table 4.** Impact of dietary supplementation of selenium on liver antioxidant enzymes and lipid peroxidation adduct (malondialdehyde, MDA) in piglets in response to diquat challenge (*n* 6) (Mean values with their standard errors; six observations per least square mean value)

Items	Diquat challenge					sem	*P*
	NC	PC	SS	SC	SY		
SOD (U/mg protein)	0·245^a^	0·124^b^	0·192^a,b^	0·244^a^	0·181^a,b^	0·043	<0·05
GPx (nmol/min per mg protein)	23·81^a^	19·98^b^	17·87^c^	20·97^b^	26·02^a^	1·59	<0·05
TAC (mmol Trolox/mg protein)	0·031	0·028	0·023	0·034	0·032	0·01	0·24
Catalase (µmol formaldehyde/min per mg protein)	170·76	281·76	173·04	223·12	210·58	36·86	0·07
MDA (µmol/mg protein)	0·943	1·136	0·822	1·011	0·875	0·69	0·09

NC, negative control; PC, positive control; SS, sodium selenite; SC, soyabean protein-chelated Se; SY, selenised yeast; SOD, superoxide dismutase; GPx, glutathione peroxidase; TAC, total antioxidant capacity; MDA, malondialdehyde.

^a,b,c^ Mean values within a row with unlike superscript letters were significantly different (*P* < 0·05).

### Liver metabolites

**Fig. 2.**
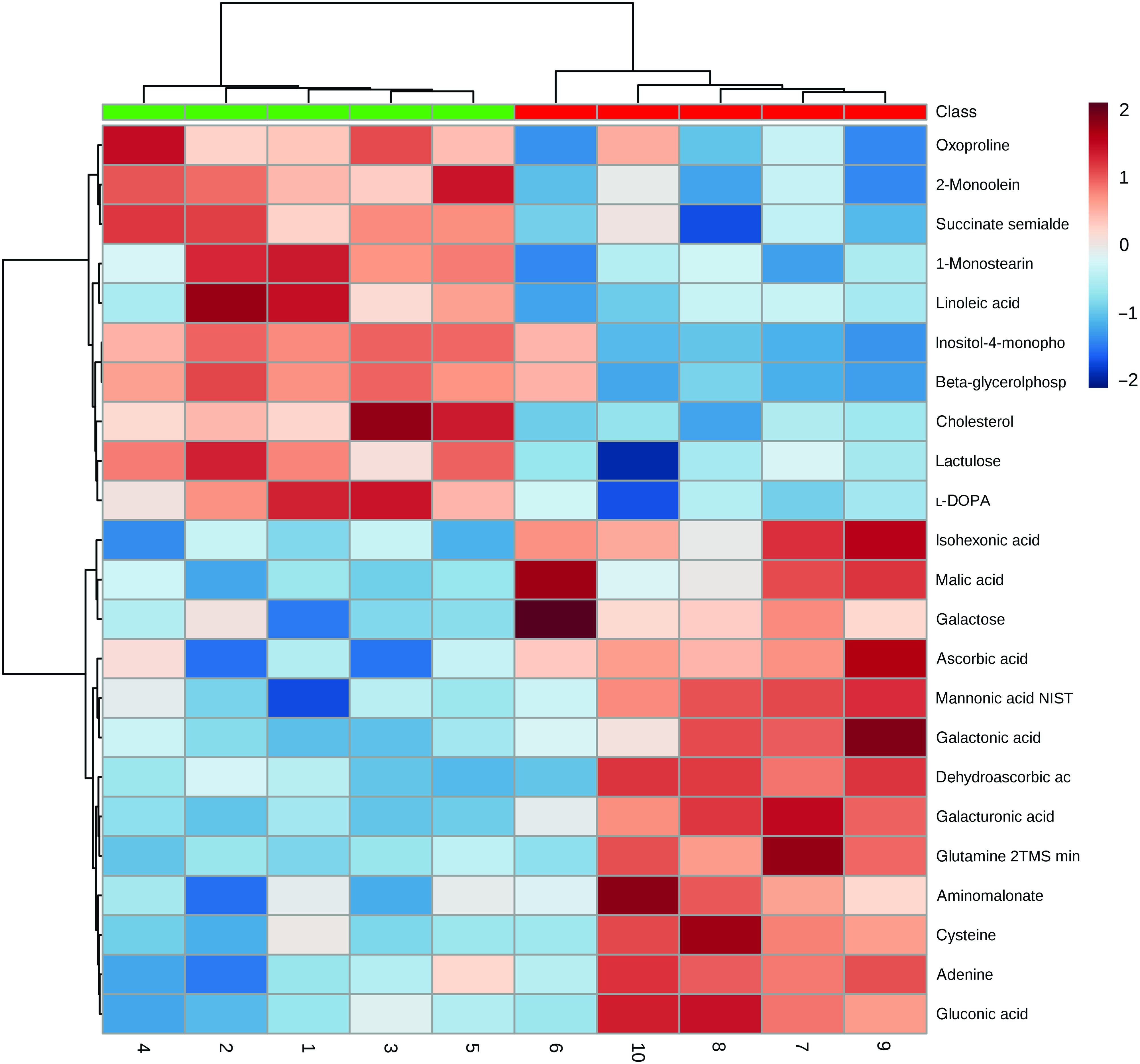
Heat map and hierarchical clustering of twenty-three hepatic metabolites significantly affected at day 7 post-diquat challenge in nursery pigs. Class: 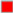, negative control without diquat challenge or dietary selenium supplementation; 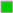, positive control with diquat challenge without dietary selenium supplementation.

**Fig. 3.**
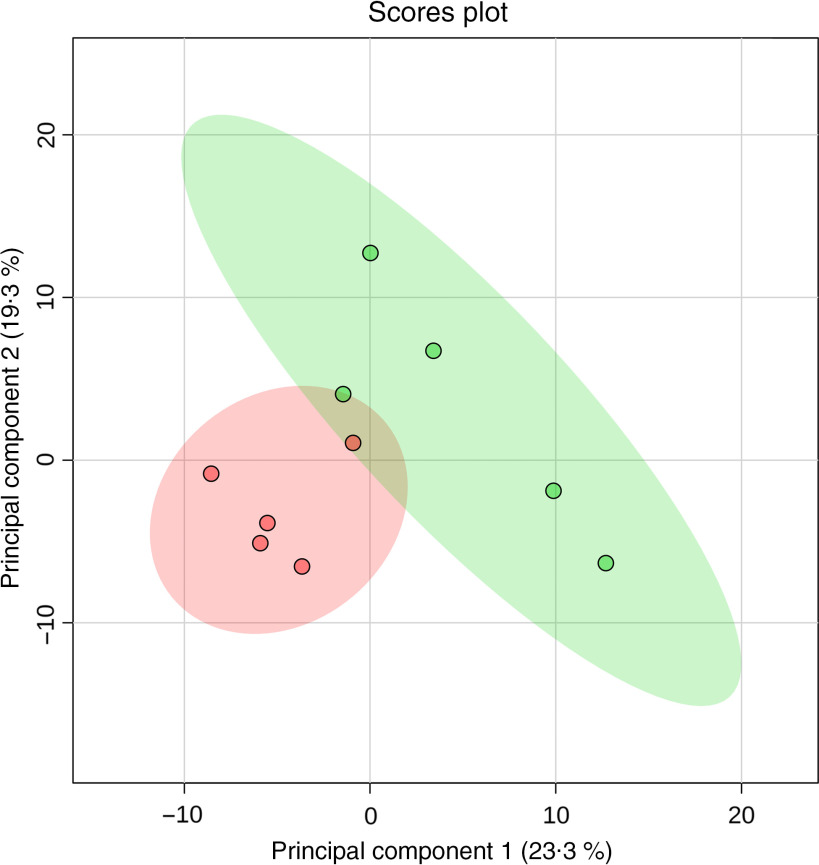
Two-dimensional score plots of principal component analysis of hepatic metabolites in post-weaning piglets on day 7 post-diquat challenge. Confidence ranges of 95 % are depicted as circled areas. Numbers listed in parentheses of each principal component indicate the variance explained. Dots indicate individual samples from the NC or PC group (*n* 5), respectively. 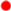, Negative control without diquat challenge or dietary selenium supplementation; 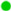, positive control with diquat challenge without dietary selenium supplementation.

In the liver, 700 metabolites were detected, of which 203 were identified. Twenty-two metabolites were significantly different between the NC and PC groups (false discovery rate < 0·15) (Table 5, Fig. 2). Hierarchical clustering analysis further revealed greater within-group resemblance in metabolite profiles in both PC and NC liver samples (Fig. 3). In PCA analysis between PC and NC, the top two components explained 42·6 % of variance. The distribution of samples was largely separated by treatment suggesting notable effect of diquat on liver metabolome. None of the hepatic metabolites was different between SY and NC or between SY and PC (data not shown). PCA revealed relatively greater similarity in distribution pattern between SY and NC (online Supplementary Fig. S1). Substantial individual variations were observed for samples from the SY group.

**Table 5.** Diquat injection altered (false discovery rate (FDR)-adjusted *P* < 0·15) twenty-three liver metabolites in post-weaning pigs

Metabolite	Log_2_FC (PC/NC)*	*P*	FDR
Inositol-4**-**monophosphate	2·06	0·001	0·046
*β*-Glycerolphosphate	1·83	0·001	0·046
Succinate semialdehyde	1·38	0·001	0·046
1-Monostearin	1·12	0·002	0·047
2-Monoolein	1·00	0·001	0·046
Linoleic acid	0·99	0·017	0·149
Levodopa	0·93	0·002	0·046
Cholesterol	0·56	0·002	0·046
5-Oxoproline	0·32	0·011	0·117
Galactose	–0·20	0·012	0·122
Aminomalonate	–0·45	0·015	0·145
Malic acid	–0·48	0·005	0·081
Adenine	–0·56	0·009	0·109
Ascorbic acid	–0·67	0·006	0·084
Isohexoic acid	–0·80	0·002	0·046
Cysteine	–1·64	0·011	0·117
Dehydroascorbic acid	–1·99	0·016	0·149
Gluconic acid	–2·21	0·009	0·109
Glutamine di(trimethylsilyl) derivative	–2·40	0·009	0·109
Galactonic acid	–2·44	0·005	0·081
Mannonic acid	–2·56	0·005	0·081
Galacturonic acid	–2·66	<0·001	0·046

* Log_2_(fold change) of metabolite in comparison between positive control (diquat injection) and negative control (saline injection). Metabolites were sorted based on Log_2_(fold change) from the largest to smallest.

## Discussion

### Effect of selenium supplements on growth performance and antioxidant capacity before diquat challenge

Adequate Se intake critically links to growth and health. Se supplementation has shown to improve growth, dietary nutrient digestibility and utilisation in young rats^(26)^ and weanling pigs^(27)^ that were previously nursed by Se-deficient dams. The underlying mechanism is not fully understood, but impairments in bone metabolism^(28)^ and dysregulated thyroid hormone^(29)^ were considered as contributing factors to Se-deficiency-induced growth retardation. In the present study, Se content in the basal diet (<0·2 mg/kg) was below recommendation on minimum Se requirement (0·3 mg/kg) for pigs weighing 5–11 kg. Lack of effect by Se supplements on growth prior to diquat challenge is probably due to a relatively short period of feeding a Se-deficient diet. Although both organic and inorganic forms of Se are efficiently absorbed, organic Se (i.e. selenomethionine and selenocysteine) showed greater tissue and cellular retention than inorganic source of Se in humans and animals^(16,30–32)^. Tissue or plasma GPx activity is a well-accepted biomarker in assessment of bioavailability of dietary Se^(10)^. Inorganic Se (i.e. selenite) is considered to be more readily converted to hydrogen selenide, the substrate for synthesis of GPx, compared with selenomethionine^(10)^. Studies comparing the effect of Se sources (organic *v*. inorganic) on GPx activity yielded inconsistent results^(12,16,33,34)^. The activity of GPx seemed to be affected by the duration and dose of Se supplements as well. Mahan *etal*.^(12)^ noted significant interaction between source of Se (selenite *v*. Se-enriched yeast) and Se level on plasma GPx activity showing that organic Se conferred greater activity at 0·2 and 0·3 mg/kg, but lower activity at 0·05 or 0·1 mg/kg compared with inorganic Se. In another study, Mahan & Parrett^(33)^ reported that finishing pigs that received selenite had greater serum GPx activity compared with those with Se-enriched yeast, when both sources of Se were supplemented at 0·1 mg/kg in a Se-deficient basal diet. However, difference in serum GPx activity was minimal when Se was supplemented at 0·3 or 0·5 mg/kg. In the present study, the plasma GPx activity was the highest in the SY group on day 0 PI, suggesting greater bioavailability of organic Se and foretelling robuster resilience to oxidative insults. Soyabean protein-chelated Se was a novel source of organic Se that was synthesised by chelating SS with hydrolysed soyabean proteins. Previously, SC has only been tested in broiler chicken in physiological state^(35)^. In that study, birds were fed a Se-deficient diet (NC), Se-replete diet (PC) or the Se-deficient diet supplemented with SC, SY or SS (same products as used in the present study)^(35)^. In comparison with the NC group, all sources of Se significantly increased Se deposition in liver and breast meat and enhanced blood GPx activity to the extent that is comparable with the PC group, while SY displayed the most prominent effect among all sources of Se^(35)^. Interestingly, the SY group had the lowest feed intake and weight gain during the 35-d feeding trial. Findings from our study were not in agreement with regard to the effect of SY on growth performance. However, in consideration of the obvious difference in animal species and experimental design, direct comparison of results between the two studies is forbidden.

### Effects of selenium supplements on resilience to diquat-induced oxidative stress

A trend of higher plasma lipid peroxidation adduct (MDA) that was observed only at 6 and 48 h PI (PC *v*. NC) suggests an acute oxidative stress in liver. Our finding is in agreement with Mao *etal*.^(22)^ wherein the same dose of diquat as used in our study induced reversible oxidative stress in weanling pigs. The drastic surge of plasma cortisol at 6 h PI (PC *v*. NC) suggested induction of acute stress. Similarly, Wang *etal*.^(7)^ also reported radical increase of stress hormones (cortisol and adrenaline) that peaked at 1·5 h and remained elevated at 15 h post-diquat injection (10 mg/kg BW) in weanling pigs. In the present study, changes in number of circulating neutrophils post-diquat injection paralleled with the changes in plasma cortisol in comparison between the PC and NC groups. Whether or not diquat has direct effect on immune cell trafficking is unknown, but there is a rich literature identifying adrenal steroid hormones as the primary mediators that modulate leucocyte profiles and distribution under stress conditions^(36)^. Through either acute stress models or direct administration, circulating corticosterone was accompanied by a rapid increase in numbers of blood neutrophils and by a decrease in numbers of circulating lymphocytes in rats^(37,38)^, whereas changes in leucocyte trafficking rapidly reversed after the cession of stress^(37)^. Therefore, changes in blood neutrophils after diquat exposure are probably mediated by stress hormones (i.e. cortisol). Dietary Se supplementation, regardless of the source of Se, attenuated the surge of cortisol and circulating neutrophils, suggesting the beneficial effect of Se on mitigating diquat-induced acute stress.

Endogenous antioxidant enzymes serve as critical defence against free radicals. GPx is responsible for removing lipid hydroperoxides. However, exaggerated production of hydrogen peroxide has been shown to inhibit cellular GPx activity^(39)^. In the present study, the lower plasma GPx activity (PC *v*. NC) at 6 and 48 h PI underscored compromised antioxidant defence and explained high plasma MDA content in the PC group. Zheng *etal*.^(40)^ also observed reduction of plasma GPx activity 7 d post-diquat injection in post-weaning pigs. Liver and kidney are the main target organs of diquat toxicity^(18,19,41)^. In contrast to acute systemic stress, hepatic GPx and SOD activities are still depressed (PC *v*. NC) on day 7 PI, suggesting a lasting effect of diquat that depleted endogenous antioxidant enzymes in liver. Our finding is consistent with another study wherein oxidative insults remained evident in liver of post-weaning pigs on day 13 post-diquat injection (10 mg/kg BW)^(7)^.

Sufficient Se supply is required to replenish cellular GPx activity in confrontation of oxidative stress. Rats fed a Se-deficient diet displayed increased vulnerability to diquat toxicity with devastating liver and kidney necrosis as compared with those fed a diet with normal Se content^(42)^. In the present study, the plasma GPx activity was the highest in the SY group post-diquat challenge. This might be partially explained by the pre-existing higher plasma GPx activity in SY before diquat challenge. Moreover, SY, but not SS or SC, restored plasma GPx activity to the level of the NC group at 6 h, on day 2 and day 7 PI and reinstated hepatic GPx activity on day 7 PI. A large percentage (>60 %) of Se in SY existed as organic form (selenomethionine)^(10)^. Studies have consistently found that organic Se associated with greater increase of plasma Se compared with inorganic Se, when both were supplemented at the same level in diets for growing or finishing pigs^(11,12,16,33)^. Plasma Se is readily mobilised for synthesis of GPx under acute oxidative insult, which possibly explained the greater GPx activity in SY pigs post-diquat injection.

Mao *etal*.^(22)^ observed reduction in plasma SOD activity in weaned pigs at day 7 post-diquat injection as the only time point measured in their study. However, the plasma SOD activity was not compromised by diquat injection in our study. The finding of enhanced SOD activity by different sources of Se since 24 h PI was intriguingly, as Se is not a known factor to regulate SOD activity. Inconsistent changes of plasma SOD activity in response to diquat and Se supplementation are enigmatic and need to be further explored.

### Dietary supplementation of selenised yeast diminished diquat-induced metabolic perturbation in liver

Acute diquat poisoning is extremely detrimental to the function of liver and kidney^(41)^. Overwhelming hepatic oxidative stress and clear signs of hepatopathology were detected in mice after diquat exposure (intraperitoneal (i.p.) injection) at a dose of 125 mg/kg^(43)^. Nevertheless, in the present study, diquat exposure (i.p. injection at 10 mg/kg) at a much lower dose increased MDA concentration in liver by approximately 20 % (PC *v*. NC), suggesting induction of hepatic lipid peroxidation. Mao *etal*.^(22)^ also observed elevated hepatic lipid peroxidation in pigs injected with the same dose of diquat as used in our study. Endogenous antioxidant enzymes act as buffer to neutralise reactive oxygen species and protect cellular lipids and proteins from oxidative damage. Reduction in activities of SOD and GPx in the liver (PC *v*. NC) highlighted that both enzymes were consumed to counteract diquat-induced redox disturbance, whereas organic Se supplements, namely SC and SY, replenished hepatic SOD and GPx, respectively.

Our study is the first to report global changes of hepatic metabolites (PC *v*. NC) in pigs in response to diquat injection (Fig. 2). As shown in the score plot of PCA analysis (Fig. 3), minimal overlapping area between samples from different treatments implies major difference in metabolic profiles between PC and NC. Relatively wide distribution of PC samples highlighted large within-group variation which is probably ascribed to individual variations in resilience to oxidative insult. Four metabolites (cysteine, oxoproline, ascorbic acid and dehydroascorbic acid) that are involved in the synthesis of cellular antioxidants (i.e. glutathione (GSH) and ascorbic acid) were altered in response to diquat challenge. Glutathione, a tripeptide that is present in relatively high concentration in mammalian cells (0·5–10 mmol/l), is readily oxidised to glutathione disulphide by free radicals and thus protects cells against oxidative stress^(44)^. Among three constituent amino acids of GSH, the intracellular pool of cysteine in rat liver was relatively small (0·15–0·25 mmol/l) compared with that of glutamate (2–4 mmol/l) and glycine (1·5–2 mmol/l)^(45)^. Cysteine availability is one of the limiting factors in GSH synthesis in humans and rats^(45–47)^. In the present study, reduction of cysteine (PC *v*. NC; Table 5) in the liver presumably foretells the depletion of intracellular GSH. 5-Oxoproline is an intermediate metabolite of *γ*-glutamyl cycling in GSH synthesis. Consumption of a diet that is devoid of cysteine and methionine significantly increased plasma flux, oxidation and urinary excretion of oxoproline in humans^(48)^, suggesting that cysteine deficiency decreased salvage of oxoproline and probably compromised GSH synthesis. In the present study, the increase of 5-oxoproline in the liver by diquat challenge is consistent with the reduction of cysteine, which collectively suggested the depletion of GSH. Ascorbic acid (AA) acts as a major free radical scavenger *in vivo* and is converted to dehydroascorbic acid (DHA) upon oxidation. Plasma concentrations of AA and DHA serve as reliable biomarkers of oxidative stress caused by smoking^(49)^. Because of its large ascorbate store, the liver is considered a reservoir that maintains the homoeostasis of plasma ascorbate^(50)^. In the present study, a lower hepatic AA content in PC compared with NC underscored consumption of the AA store in response to diquat-induced oxidative insult. Under physiological conditions, intracellular DHA is instantly reduced to regenerate AA at the expense of cellular reductants (i.e. NADH, NADPH or GSH)^(51)^. The reaction is primarily catalysed by NADPH-dependent thioredoxin reductase and, to a lesser extent, by glutathione-dependent DHA reductase^(51,52)^. As a selenoprotein, thioredoxin reductase activity in the liver is depressed in rats fed a Se-deficient diet^(52)^. However, in the presence of H_2_O_2_, DHA is readily hydrolysed and undergoes irreversible degradation *in vitro*, resulting in a loss of ascorbic acid^(53)^. Therefore, reduction in both AA and DHA (PC *v*. NC) presumably resulted from the depletion of cellular reductants and conditional Se deficiency under acute oxidative insult.

The finding of a unanimous increase of lipid metabolites (*β*-glycerolphosphate, 1-monostearin, 2-monoolein, linoleic acid and cholesterol) (PC *v*. NC) highlighted an overall effect of diquat on hepatic lipid metabolism. Cell membrane lipids are extremely vulnerable to oxidative damage, while diquat exposure has shown to cause lipid peroxidation and liver necrosis^(54)^. It is plausible that increased lipid metabolites were derived from decomposition of cellular membranes. In addition, oxidative stress hypothetically impairs mitochondrial ATP production and subsequently triggers *β*-oxidation of cellular lipids, which might contribute to changes of lipid metabolites as well. A recent study with transcriptome analysis revealed the significant changes in gene sets associated with energy reserve and cellular lipid metabolic processes in the liver of diquat-challenged pigs^(55)^. Therefore, changes in lipid metabolites as observed in our study could also be mediated through transcriptional modulation of related genes. Furthermore, Seo *et al.* ^(56)^ reported that treatment with H_2_O_2_ significantly increased the expression of genes related to cholesterol synthesis in HepG2 cells (a human liver cancer cell line), explaining the increase of cholesterol content in the liver of diquat-challenged pigs in our study.

In contrast to the increase of lipid metabolites, the unanimous reduction of galactose and sugar derivatives (gluconic acid, galactonic acid and galacturonic acid) in PC may be partially attributed to lower feed intake, as lactose accounted for 6 % of the diet. Unlike humans, pigs have functional l-gulonolactone oxidase in the liver^(57)^ and thus can utilise glucose, galactose and oxidised sugar acids (i.e. gluconic acid, galactonic acid and galacturonic acid) as substrates for *de novo* synthesis of ascorbic acid^(58)^. The lower hepatic ascorbic acid content (PC *v*. NC), therefore, may be due in part to substrate deficiency because of low feed intake under oxidative stress. This might further aggravate diquat-induced oxidative stress in liver.

In our previous discussion, supplementation of SY was more effective in mitigating diquat-induced oxidative stress. Thus, only liver samples of the SY group were submitted for metabolome analysis. In agreement with the results of other analyses (i.e. oxidative measurements in blood and liver), SY partially recuperated diquat-induced metabolic perturbation in the liver (online Supplementary Fig. S1). This is supported by the fact that the 95 % confidence zone of SY was largely aligned and overlapped with that of NC rather than PC samples in the score plot of PCA analysis. However, a substantial within-group variation was also detected among SY samples and hampers detection of statistical difference for any metabolites that were altered by diquat challenge. The inconsistency in hepatic metabolome of SY samples may partially ascribe to the relatively large individual variations in post-challenge feed intake (Fig. 1), which may affect Se intake and thus affect susceptibility to diquat toxicity (Fig. 3). Future study with a larger sample size is warranted.

In conclusion, the present experiment confirms that intraperitoneal injection of diquat could induce acute oxidative stress and systemic inflammation, which provides a valuable pig model to investigate the mechanistic importance of exogenous antioxidants, such as Se, in human and animal health. The results also highlighted that supplementation of 0·3 mg/kg Se as Se-enriched yeast could prime the systemic antioxidant capacity and subsequently enhance resilience to diquat-induced oxidative stress and inflammation in weaned pigs. Diquat challenge altered lipid and galactose metabolism and reduction in metabolites associated with cellular antioxidant synthesis. However, the effect of SY on hepatic metabolome was not congruent among all samples emphasising the necessity of a larger sample size in future study. Neither SS nor SC displayed consistent antioxidant effects that is comparable with SY in battle against diquat-induced oxidative stress in weaned pigs.

## Supplementary material

For supplementary material accompanying this paper visit http://dx.doi.org/10.1017/S0007114520000689. click here to view supplementary material
