# Supplementary material for: Organic selenium supplement partially alleviated diquat-induced oxidative insults and hepatic metabolic stress in nursery pigs
Source: Br J Nutr. 2020 Mar 2;124(1):23–33. doi: 10.1017/S0007114520000689 (PMC7512145; doi:10.1017/S0007114520000689)
Supplement: Supplementary file 1 [file S0007114520000689sup.zip › S0007114520000689sup002.docx]

**Table S1.** Difference in abundance of mapped hepatic metabolites between positive control and negative control group (PC/NC)

| Compound name | Fold Change | log2(FC) | p.value | FDR |
| --- | --- | --- | --- | --- |
| 2-monoolein | 2.00 | 1.00 | 0.001 | 0.046 |
| beta-glycerolphosphate | 3.55 | 1.83 | 0.001 | 0.046 |
| cholesterol | 1.47 | 0.56 | 0.002 | 0.046 |
| galacturonic acid | 0.16 | -2.66 | 0.000 | 0.046 |
| inositol-4-monophosphate | 4.16 | 2.06 | 0.001 | 0.046 |
| isohexonic acid | 0.57 | -0.80 | 0.002 | 0.046 |
| lactulose | 3.34 | 1.74 | 0.002 | 0.046 |
| L-DOPA | 1.90 | 0.93 | 0.002 | 0.046 |
| succinate semialdehyde | 2.60 | 1.38 | 0.001 | 0.046 |
| 1-monostearin | 2.17 | 1.12 | 0.002 | 0.047 |
| galactonic acid | 0.18 | -2.44 | 0.005 | 0.081 |
| malic acid | 0.72 | -0.48 | 0.005 | 0.081 |
| mannonic acid NIST | 0.17 | -2.56 | 0.005 | 0.081 |
| ascorbic acid | 0.63 | -0.67 | 0.006 | 0.084 |
| adenine | 0.68 | -0.56 | 0.009 | 0.109 |
| gluconic acid | 0.22 | -2.21 | 0.009 | 0.109 |
| glutamine 2TMS minor | 0.19 | -2.40 | 0.009 | 0.109 |
| cysteine | 0.32 | -1.64 | 0.011 | 0.117 |
| oxoproline | 1.25 | 0.32 | 0.011 | 0.117 |
| galactose | 0.87 | -0.20 | 0.012 | 0.122 |
| aminomalonate | 0.73 | -0.45 | 0.015 | 0.145 |
| dehydroascorbic acid | 0.25 | -1.99 | 0.016 | 0.149 |
| linoleic acid | 1.99 | 0.99 | 0.017 | 0.149 |
| lactic acid | 0.56 | -0.83 | 0.020 | 0.161 |
| ornithine | 0.54 | -0.90 | 0.020 | 0.161 |
| pentonic acid | 0.58 | -0.79 | 0.021 | 0.161 |
| 1-methylinosine NIST | 1.67 | 0.74 | 0.022 | 0.163 |
| oleic acid | 1.44 | 0.52 | 0.025 | 0.163 |
| phosphoethanolamine | 3.16 | 1.66 | 0.024 | 0.163 |
| spermine | 0.47 | -1.09 | 0.025 | 0.163 |
| squalene | 2.42 | 1.27 | 0.024 | 0.163 |
| butyrolactam NIST | 0.75 | -0.42 | 0.028 | 0.175 |
| ethanolamine | 2.22 | 1.15 | 0.029 | 0.178 |
| urea | 3.27 | 1.71 | 0.033 | 0.196 |
| lysine | 0.66 | -0.59 | 0.035 | 0.201 |
| zymosterol | 0.54 | -0.89 | 0.037 | 0.210 |
| hexuronic acid | 0.73 | -0.45 | 0.041 | 0.225 |
| glutaric acid | 1.58 | 0.66 | 0.044 | 0.233 |
| oxamic acid | 1.54 | 0.62 | 0.045 | 0.233 |
| 2,5-dihydroxypyrazine NIST | 1.93 | 0.95 | 0.048 | 0.244 |
| docosahexaenoic acid | 1.29 | 0.37 | 0.051 | 0.253 |
| arachidonic acid | 1.64 | 0.71 | 0.060 | 0.289 |
| 4-hydroxybutyric acid | 0.68 | -0.55 | 0.063 | 0.291 |
| lactamide | 1.51 | 0.59 | 0.063 | 0.291 |
| asparagine | 0.77 | -0.38 | 0.069 | 0.310 |
| heptadecanoic acid | 0.59 | -0.77 | 0.076 | 0.322 |
| nicotinamide | 1.37 | 0.45 | 0.076 | 0.322 |
| oleamide NIST | 0.43 | -1.22 | 0.076 | 0.322 |
| 4-aminobutyric acid | 0.81 | -0.31 | 0.090 | 0.373 |
| acetophenone NIST | 0.56 | -0.83 | 0.095 | 0.377 |
| sophorose | 1.44 | 0.53 | 0.095 | 0.377 |
| dodecanol | 1.26 | 0.34 | 0.098 | 0.380 |
| pinitol | 1.64 | 0.71 | 0.099 | 0.380 |
| glucose-1-phosphate | 0.82 | -0.28 | 0.103 | 0.387 |
| glycerol-alpha-phosphate | 1.23 | 0.29 | 0.116 | 0.418 |
| hexitol | 1.47 | 0.56 | 0.116 | 0.418 |
| hydroxycarbamate NIST | 0.31 | -1.68 | 0.122 | 0.418 |
| pseudo uridine | 1.45 | 0.54 | 0.118 | 0.418 |
| tyrosine | 0.82 | -0.28 | 0.120 | 0.418 |
| O-phosphoserine | 1.60 | 0.68 | 0.141 | 0.478 |
| galactose-6-phosphate | 0.64 | -0.64 | 0.144 | 0.480 |
| conduritol-beta-epoxide | 0.84 | -0.25 | 0.149 | 0.489 |
| fructose | 1.45 | 0.54 | 0.160 | 0.515 |
| pyruvic acid | 0.77 | -0.38 | 0.166 | 0.527 |
| 3,6-anhydro-D-galactose | 1.27 | 0.35 | 0.175 | 0.531 |
| isothreonic acid | 1.44 | 0.53 | 0.174 | 0.531 |
| putrescine | 1.77 | 0.82 | 0.175 | 0.531 |
| uracil | 1.47 | 0.56 | 0.180 | 0.537 |
| 2-monopalmitin | 1.26 | 0.33 | 0.183 | 0.539 |
| 1-monoolein | 0.58 | -0.78 | 0.202 | 0.574 |
| beta-sitosterol | 1.27 | 0.35 | 0.210 | 0.574 |
| cis-gondoic acid | 1.37 | 0.45 | 0.207 | 0.574 |
| epsilon-caprolactam | 1.24 | 0.31 | 0.209 | 0.574 |
| hydroquinone | 0.69 | -0.54 | 0.218 | 0.574 |
| hydroxylamine | 0.32 | -1.64 | 0.212 | 0.574 |
| myristic acid | 0.82 | -0.28 | 0.216 | 0.574 |
| p-tolyl glucuronide | 1.92 | 0.94 | 0.199 | 0.574 |
| salicylic acid | 1.27 | 0.34 | 0.222 | 0.577 |
| arabinose | 1.32 | 0.40 | 0.229 | 0.583 |
| glycolic acid | 0.82 | -0.28 | 0.230 | 0.583 |
| alanine | 0.78 | -0.36 | 0.252 | 0.598 |
| cytidine-5-monophosphate | 1.26 | 0.34 | 0.252 | 0.598 |
| hexose | 1.37 | 0.45 | 0.253 | 0.598 |
| histidine | 0.55 | -0.87 | 0.249 | 0.598 |
| threonic acid | 1.36 | 0.45 | 0.250 | 0.598 |
| UDP-N-acetylglucosamine | 1.24 | 0.31 | 0.253 | 0.598 |
| 1,3,5-trimethylcyanuric acid | 1.19 | 0.25 | 0.267 | 0.613 |
| inosine | 1.26 | 0.33 | 0.269 | 0.613 |
| N-carbamoylaspartate | 0.77 | -0.37 | 0.268 | 0.613 |
| levoglucosan | 0.75 | -0.41 | 0.272 | 0.614 |
| leucine | 1.25 | 0.33 | 0.278 | 0.618 |
| tryptophan | 0.87 | -0.20 | 0.280 | 0.618 |
| pyrrole-2-carboxylic acid | 0.88 | -0.19 | 0.286 | 0.624 |
| beta-alanine | 1.10 | 0.13 | 0.296 | 0.639 |
| 1,2-anhydro-myo-inositol NIST | 0.80 | -0.31 | 0.309 | 0.647 |
| adenosine | 1.37 | 0.45 | 0.312 | 0.647 |
| glyceric acid | 1.65 | 0.72 | 0.307 | 0.647 |
| lyxitol | 1.31 | 0.39 | 0.306 | 0.647 |
| fumaric acid | 0.85 | -0.24 | 0.319 | 0.647 |
| glucose | 0.81 | -0.30 | 0.317 | 0.647 |
| citrulline | 1.19 | 0.25 | 0.328 | 0.653 |
| uric acid | 0.67 | -0.57 | 0.326 | 0.653 |
| hypoxanthine | 1.39 | 0.47 | 0.338 | 0.661 |
| threonine | 0.88 | -0.19 | 0.335 | 0.661 |
| 1-monoheptadecanoyl glyceride NIST | 0.83 | -0.27 | 0.383 | 0.690 |
| 65h-benzocphenanthridinone, 11,12-dihydro- NIST | 0.87 | -0.21 | 0.359 | 0.690 |
| arabitol | 2.74 | 1.46 | 0.381 | 0.690 |
| cystine | 1.42 | 0.50 | 0.373 | 0.690 |
| hexose-6-phosphate | 0.85 | -0.23 | 0.377 | 0.690 |
| myo-inositol | 1.22 | 0.29 | 0.377 | 0.690 |
| palmitic acid | 0.84 | -0.24 | 0.384 | 0.690 |
| xanthine | 1.23 | 0.29 | 0.374 | 0.690 |
| xylose | 1.12 | 0.16 | 0.368 | 0.690 |
| uridine | 0.79 | -0.33 | 0.400 | 0.712 |
| xanthosine | 1.13 | 0.17 | 0.406 | 0.716 |
| proline | 1.26 | 0.33 | 0.411 | 0.719 |
| isomaltose | 1.22 | 0.29 | 0.417 | 0.723 |
| orotic acid | 1.19 | 0.25 | 0.420 | 0.723 |
| 1-hexadecanol | 0.82 | -0.28 | 0.452 | 0.734 |
| behenic acid | 1.16 | 0.21 | 0.450 | 0.734 |
| fructose-1-phosphate | 0.74 | -0.44 | 0.458 | 0.734 |
| fructose-6-phosphate | 0.86 | -0.21 | 0.456 | 0.734 |
| galactinol | 1.33 | 0.41 | 0.435 | 0.734 |
| maltotriose | 1.33 | 0.41 | 0.452 | 0.734 |
| pantothenic acid | 1.33 | 0.41 | 0.459 | 0.734 |
| sucrose | 1.13 | 0.18 | 0.441 | 0.734 |
| valine | 1.23 | 0.30 | 0.440 | 0.734 |
| pelargonic acid | 0.90 | -0.15 | 0.466 | 0.734 |
| ribose | 1.21 | 0.28 | 0.466 | 0.734 |
| enolpyruvate NIST | 1.12 | 0.16 | 0.471 | 0.735 |
| arachidic acid | 1.19 | 0.25 | 0.489 | 0.758 |
| pyrophosphate | 0.95 | -0.08 | 0.505 | 0.770 |
| ribulose-5-phosphate | 0.93 | -0.11 | 0.504 | 0.770 |
| 1,2,4-benzenetriol | 0.93 | -0.11 | 0.516 | 0.777 |
| dehydroabietic acid | 1.29 | 0.37 | 0.536 | 0.777 |
| N-acetylaspartic acid | 1.01 | 0.02 | 0.518 | 0.777 |
| n-acetyl-d-hexosamine | 1.32 | 0.41 | 0.528 | 0.777 |
| propane-1,3-diol NIST | 0.94 | -0.09 | 0.539 | 0.777 |
| ribose-5-phosphate | 1.20 | 0.26 | 0.538 | 0.777 |
| sorbitol | 0.98 | -0.03 | 0.531 | 0.777 |
| stearic acid | 0.90 | -0.15 | 0.537 | 0.777 |
| glycerol | 0.96 | -0.06 | 0.544 | 0.778 |
| chlorogenic acid | 1.21 | 0.28 | 0.564 | 0.784 |
| creatinine | 1.77 | 0.82 | 0.557 | 0.784 |
| glucose-6-phosphate | 0.97 | -0.05 | 0.553 | 0.784 |
| lanosterol | 0.92 | -0.12 | 0.561 | 0.784 |
| 2-hydroxyglutaric acid | 1.14 | 0.19 | 0.609 | 0.784 |
| 5-methoxytryptamine | 0.75 | -0.42 | 0.605 | 0.784 |
| alpha-aminoadipic acid | 2.81 | 1.49 | 0.627 | 0.784 |
| capric acid | 1.10 | 0.14 | 0.613 | 0.784 |
| cellobiose | 1.09 | 0.13 | 0.598 | 0.784 |
| D-erythro-sphingosine | 1.15 | 0.20 | 0.580 | 0.784 |
| erythronic acid lactone | 0.97 | -0.04 | 0.626 | 0.784 |
| glycine | 1.05 | 0.07 | 0.581 | 0.784 |
| guanidinosuccinate | 3.80 | 1.93 | 0.634 | 0.784 |
| guanine | 1.12 | 0.17 | 0.595 | 0.784 |
| maleimide | 0.94 | -0.10 | 0.585 | 0.784 |
| methionine sulfoxide | 0.89 | -0.17 | 0.622 | 0.784 |
| palmitoleic acid | 1.20 | 0.26 | 0.633 | 0.784 |
| phosphate | 1.22 | 0.29 | 0.615 | 0.784 |
| serine | 1.12 | 0.16 | 0.602 | 0.784 |
| tocopherol alpha- | 1.14 | 0.19 | 0.631 | 0.784 |
| UDP-glucuronic acid | 0.56 | -0.83 | 0.625 | 0.784 |
| xylitol | 0.95 | -0.07 | 0.621 | 0.784 |
| citric acid | 0.78 | -0.35 | 0.648 | 0.795 |
| thymidine | 0.72 | -0.47 | 0.650 | 0.795 |
| cerotinic acid | 0.99 | -0.02 | 0.666 | 0.809 |
| fucose | 1.12 | 0.16 | 0.685 | 0.828 |
| 3-aminoisobutyric acid | 1.05 | 0.08 | 0.692 | 0.831 |
| taurine | 7.20 | 2.85 | 0.713 | 0.851 |
| 3-hydroxy-3-methylglutaric acid | 1.00 | 0.01 | 0.730 | 0.862 |
| lactitol | 1.01 | 0.01 | 0.727 | 0.862 |
| tartaric acid | 0.94 | -0.09 | 0.747 | 0.876 |
| methionine | 0.91 | -0.14 | 0.753 | 0.879 |
| ketohexose | 1.01 | 0.02 | 0.781 | 0.906 |
| 3-phosphoglycerate | 0.93 | -0.10 | 0.800 | 0.922 |
| mannose | 1.11 | 0.15 | 0.806 | 0.924 |
| aspartic acid | 0.96 | -0.05 | 0.828 | 0.942 |
| ethanol phosphate NIST | 1.02 | 0.03 | 0.844 | 0.942 |
| lyxose | 1.06 | 0.08 | 0.842 | 0.942 |
| pentadecanoic acid | 0.95 | -0.07 | 0.836 | 0.942 |
| trans-4-hydroxy-L-proline | 1.02 | 0.03 | 0.835 | 0.942 |
| glycocyamine | 0.72 | -0.48 | 0.857 | 0.951 |
| 5'-deoxy-5'-methylthioadenosine | 1.02 | 0.02 | 0.891 | 0.967 |
| glycerol-3-galactoside | 1.00 | 0.00 | 0.877 | 0.967 |
| phenylalanine | 1.04 | 0.05 | 0.884 | 0.967 |
| ribonic acid | 0.89 | -0.16 | 0.889 | 0.967 |
| 2-hydroxypyrazinyl-2-propenoic acid ethyl ester NIST | 1.05 | 0.06 | 0.913 | 0.969 |
| 5-aminovaleric acid | 1.02 | 0.03 | 0.945 | 0.969 |
| adenosine-5-monophosphate | 0.98 | -0.04 | 0.939 | 0.969 |
| guanosine | 0.97 | -0.04 | 0.937 | 0.969 |
| isoleucine | 0.99 | -0.02 | 0.921 | 0.969 |
| mannitol | 1.02 | 0.04 | 0.924 | 0.969 |
| methanolphosphate | 1.09 | 0.12 | 0.942 | 0.969 |
| octadecanol | 1.00 | 0.00 | 0.933 | 0.969 |
| succinic acid | 0.95 | -0.07 | 0.932 | 0.969 |
| tagatose | 1.07 | 0.10 | 0.930 | 0.969 |
| xylonolactone NIST | 1.02 | 0.03 | 0.901 | 0.969 |
| benzoic acid | 1.04 | 0.05 | 0.954 | 0.970 |
| oxalic acid | 1.03 | 0.04 | 0.956 | 0.970 |
| 2-aminobutyric acid | 1.09 | 0.13 | 0.973 | 0.983 |
| cholesterone | 1.01 | 0.01 | 0.985 | 0.990 |
| glutamic acid | 1.02 | 0.02 | 0.997 | 0.997 |

**Table S2.** Difference in abundance of mapped hepatic metabolites between negative control and Selenized yeast group (NC/SY)

| Compound name | Fold Change | log2(FC) | p.value | FDR |
| --- | --- | --- | --- | --- |
| 4-aminobutyric acid | 1.27 | 0.35 | 0.013 | 0.832 |
| cerotinic acid | 1.67 | 0.74 | 0.013 | 0.832 |
| linoleic acid | 0.71 | -0.49 | 0.014 | 0.832 |
| uric acid | 2.22 | 1.15 | 0.016 | 0.832 |
| 1,2-anhydro-myo-inositol NIST | 1.20 | 0.26 | 0.286 | 0.975 |
| 1-hexadecanol | 1.15 | 0.20 | 0.573 | 0.975 |
| 1-methylinosine NIST | 0.88 | -0.18 | 0.441 | 0.975 |
| 1-monoheptadecanoyl glyceride NIST | 1.26 | 0.33 | 0.228 | 0.975 |
| 1-monoolein | 1.90 | 0.92 | 0.197 | 0.975 |
| 2,5-dihydroxypyrazine NIST | 0.60 | -0.73 | 0.046 | 0.975 |
| 2-aminobutyric acid | 1.72 | 0.78 | 0.161 | 0.975 |
| 2-hydroxyglutaric acid | 1.10 | 0.14 | 0.517 | 0.975 |
| 2-monoolein | 0.75 | -0.42 | 0.176 | 0.975 |
| 3,6-anhydro-D-galactose | 0.90 | -0.16 | 0.485 | 0.975 |
| 3-aminoisobutyric acid | 0.92 | -0.12 | 0.555 | 0.975 |
| 3-hydroxy-3-methylglutaric acid | 1.42 | 0.50 | 0.187 | 0.975 |
| 3-phosphoglycerate | 0.85 | -0.23 | 0.463 | 0.975 |
| 4-hydroxybutyric acid | 1.20 | 0.26 | 0.398 | 0.975 |
| 5'-deoxy-5'-methylthioadenosine | 1.19 | 0.25 | 0.396 | 0.975 |
| adenosine | 0.69 | -0.53 | 0.128 | 0.975 |
| aminomalonate | 1.14 | 0.19 | 0.289 | 0.975 |
| ascorbic acid | 0.53 | -0.91 | 0.205 | 0.975 |
| asparagine | 1.08 | 0.11 | 0.473 | 0.975 |
| aspartic acid | 0.74 | -0.44 | 0.388 | 0.975 |
| beta-alanine | 1.07 | 0.09 | 0.277 | 0.975 |
| beta-glycerolphosphate | 0.69 | -0.53 | 0.343 | 0.975 |
| beta-sitosterol | 0.85 | -0.24 | 0.277 | 0.975 |
| butyrolactam NIST | 1.23 | 0.30 | 0.071 | 0.975 |
| capric acid | 1.10 | 0.14 | 0.331 | 0.975 |
| cholesterol | 0.91 | -0.14 | 0.346 | 0.975 |
| cholesterone | 1.04 | 0.06 | 0.577 | 0.975 |
| cis-gondoic acid | 1.15 | 0.20 | 0.367 | 0.975 |
| citric acid | 1.31 | 0.39 | 0.526 | 0.975 |
| cystine | 0.75 | -0.41 | 0.148 | 0.975 |
| dehydroabietic acid | 1.07 | 0.10 | 0.501 | 0.975 |
| D-erythro-sphingosine | 0.90 | -0.15 | 0.526 | 0.975 |
| docosahexaenoic acid | 0.88 | -0.19 | 0.439 | 0.975 |
| dodecanol | 1.11 | 0.14 | 0.411 | 0.975 |
| enolpyruvate NIST | 0.89 | -0.17 | 0.433 | 0.975 |
| epsilon-caprolactam | 1.17 | 0.23 | 0.203 | 0.975 |
| erythronic acid lactone | 0.76 | -0.39 | 0.090 | 0.975 |
| fucose | 1.06 | 0.08 | 0.496 | 0.975 |
| fumaric acid | 1.09 | 0.13 | 0.523 | 0.975 |
| galactinol | 0.64 | -0.65 | 0.496 | 0.975 |
| galactonic acid | 1.27 | 0.34 | 0.559 | 0.975 |
| galactose | 1.11 | 0.16 | 0.086 | 0.975 |
| galactose-6-phosphate | 0.67 | -0.57 | 0.485 | 0.975 |
| gluconic acid | 1.65 | 0.72 | 0.463 | 0.975 |
| glucose | 1.17 | 0.23 | 0.346 | 0.975 |
| glucose-1-phosphate | 1.14 | 0.18 | 0.272 | 0.975 |
| glyceric acid | 0.89 | -0.16 | 0.531 | 0.975 |
| glycerol | 1.20 | 0.26 | 0.300 | 0.975 |
| glycerol-alpha-phosphate | 0.85 | -0.23 | 0.129 | 0.975 |
| glycine | 1.11 | 0.15 | 0.261 | 0.975 |
| guanine | 1.06 | 0.09 | 0.539 | 0.975 |
| heptadecanoic acid | 1.13 | 0.18 | 0.458 | 0.975 |
| hexitol | 0.88 | -0.19 | 0.441 | 0.975 |
| hydroquinone | 1.30 | 0.38 | 0.401 | 0.975 |
| hypoxanthine | 0.85 | -0.23 | 0.576 | 0.975 |
| inositol-4-monophosphate | 0.65 | -0.63 | 0.561 | 0.975 |
| isoleucine | 1.18 | 0.24 | 0.253 | 0.975 |
| isothreonic acid | 0.84 | -0.25 | 0.137 | 0.975 |
| ketohexose | 0.82 | -0.28 | 0.474 | 0.975 |
| lactic acid | 1.16 | 0.22 | 0.445 | 0.975 |
| lanosterol | 1.19 | 0.25 | 0.184 | 0.975 |
| L-DOPA | 0.79 | -0.34 | 0.202 | 0.975 |
| levoglucosan | 1.36 | 0.44 | 0.161 | 0.975 |
| lysine | 1.49 | 0.58 | 0.037 | 0.975 |
| lyxose | 0.88 | -0.19 | 0.466 | 0.975 |
| maleimide | 1.06 | 0.09 | 0.570 | 0.975 |
| malic acid | 1.16 | 0.22 | 0.250 | 0.975 |
| methanolphosphate | 0.93 | -0.10 | 0.509 | 0.975 |
| methionine | 1.33 | 0.41 | 0.322 | 0.975 |
| methionine sulfoxide | 1.13 | 0.18 | 0.512 | 0.975 |
| myo-inositol | 0.83 | -0.26 | 0.335 | 0.975 |
| n-acetyl-d-hexosamine | 1.23 | 0.30 | 0.544 | 0.975 |
| N-carbamoylaspartate | 1.21 | 0.27 | 0.278 | 0.975 |
| oleic acid | 0.88 | -0.19 | 0.196 | 0.975 |
| O-phosphoserine | 0.58 | -0.79 | 0.327 | 0.975 |
| ornithine | 1.47 | 0.56 | 0.183 | 0.975 |
| orotic acid | 1.08 | 0.11 | 0.535 | 0.975 |
| oxalic acid | 0.93 | -0.11 | 0.575 | 0.975 |
| oxamic acid | 0.91 | -0.14 | 0.425 | 0.975 |
| oxoproline | 0.91 | -0.14 | 0.301 | 0.975 |
| pelargonic acid | 0.70 | -0.52 | 0.488 | 0.975 |
| pentonic acid | 0.71 | -0.49 | 0.225 | 0.975 |
| phenylalanine | 1.10 | 0.14 | 0.372 | 0.975 |
| phosphoethanolamine | 0.44 | -1.17 | 0.098 | 0.975 |
| pinitol | 0.83 | -0.27 | 0.297 | 0.975 |
| proline | 1.09 | 0.13 | 0.411 | 0.975 |
| propane-1,3-diol NIST | 1.14 | 0.18 | 0.170 | 0.975 |
| p-tolyl glucuronide | 0.80 | -0.32 | 0.438 | 0.975 |
| pyrophosphate | 0.90 | -0.16 | 0.330 | 0.975 |
| pyrrole-2-carboxylic acid | 1.18 | 0.24 | 0.093 | 0.975 |
| pyruvic acid | 1.05 | 0.08 | 0.526 | 0.975 |
| ribonic acid | 0.62 | -0.70 | 0.279 | 0.975 |
| ribose | 1.19 | 0.25 | 0.440 | 0.975 |
| ribose-5-phosphate | 0.47 | -1.08 | 0.325 | 0.975 |
| ribulose-5-phosphate | 1.14 | 0.19 | 0.472 | 0.975 |
| sorbitol | 1.10 | 0.14 | 0.552 | 0.975 |
| spermine | 1.46 | 0.54 | 0.274 | 0.975 |
| squalene | 0.64 | -0.65 | 0.125 | 0.975 |
| sucrose | 1.44 | 0.52 | 0.536 | 0.975 |
| tagatose | 1.25 | 0.32 | 0.215 | 0.975 |
| tartaric acid | 0.79 | -0.33 | 0.387 | 0.975 |
| taurine | 0.73 | -0.46 | 0.532 | 0.975 |
| threonic acid | 0.78 | -0.35 | 0.093 | 0.975 |
| threonine | 1.47 | 0.56 | 0.025 | 0.975 |
| thymidine | 1.48 | 0.56 | 0.528 | 0.975 |
| tocopherol alpha- | 0.88 | -0.19 | 0.415 | 0.975 |
| trans-4-hydroxy-L-proline | 1.32 | 0.40 | 0.474 | 0.975 |
| tryptophan | 1.25 | 0.33 | 0.092 | 0.975 |
| tyrosine | 1.18 | 0.24 | 0.147 | 0.975 |
| uracil | 1.28 | 0.36 | 0.199 | 0.975 |
| urea | 0.70 | -0.51 | 0.273 | 0.975 |
| valine | 1.10 | 0.14 | 0.404 | 0.975 |
| xanthosine | 1.05 | 0.07 | 0.551 | 0.975 |
| xylitol | 1.09 | 0.13 | 0.501 | 0.975 |
| xylose | 0.90 | -0.16 | 0.556 | 0.975 |
| zymosterol | 1.56 | 0.64 | 0.064 | 0.975 |
| adenine | 1.07 | 0.10 | 0.591 | 0.991 |
| arabinose | 1.08 | 0.11 | 0.597 | 0.993 |
| 1,2,4-benzenetriol | 1.05 | 0.07 | 0.621 | 0.999 |
| 1,3,5-trimethylcyanuric acid | 1.01 | 0.01 | 0.786 | 0.999 |
| 1-monostearin | 0.96 | -0.06 | 0.868 | 0.999 |
| 2-hydroxypyrazinyl-2-propenoic acid ethyl ester NIST | 0.94 | -0.08 | 0.719 | 0.999 |
| 2-monopalmitin | 0.99 | -0.02 | 0.999 | 0.999 |
| 5-aminovaleric acid | 1.03 | 0.04 | 0.916 | 0.999 |
| 5-methoxytryptamine | 1.03 | 0.04 | 0.684 | 0.999 |
| 65h-benzocphenanthridinone, 11,12-dihydro- NIST | 1.01 | 0.01 | 0.947 | 0.999 |
| acetophenone NIST | 1.16 | 0.22 | 0.719 | 0.999 |
| adenosine-5-monophosphate | 0.81 | -0.31 | 0.769 | 0.999 |
| alanine | 0.99 | -0.02 | 0.967 | 0.999 |
| alpha-aminoadipic acid | 1.58 | 0.66 | 0.801 | 0.999 |
| arabitol | 1.45 | 0.54 | 0.781 | 0.999 |
| arachidic acid | 1.27 | 0.34 | 0.845 | 0.999 |
| arachidonic acid | 0.95 | -0.07 | 0.768 | 0.999 |
| behenic acid | 1.10 | 0.14 | 0.680 | 0.999 |
| benzoic acid | 0.99 | -0.02 | 0.946 | 0.999 |
| cellobiose | 0.95 | -0.07 | 0.766 | 0.999 |
| chlorogenic acid | 1.17 | 0.23 | 0.981 | 0.999 |
| citrulline | 1.01 | 0.02 | 0.843 | 0.999 |
| conduritol-beta-epoxide | 0.95 | -0.08 | 0.994 | 0.999 |
| creatinine | 1.03 | 0.05 | 0.848 | 0.999 |
| cysteine | 1.43 | 0.52 | 0.622 | 0.999 |
| cytidine-5-monophosphate | 1.10 | 0.13 | 0.925 | 0.999 |
| dehydroascorbic acid | 0.97 | -0.04 | 0.735 | 0.999 |
| ethanol phosphate NIST | 0.98 | -0.03 | 0.917 | 0.999 |
| ethanolamine | 0.86 | -0.22 | 0.961 | 0.999 |
| fructose | 1.02 | 0.03 | 0.962 | 0.999 |
| fructose-1-phosphate | 0.98 | -0.03 | 0.806 | 0.999 |
| fructose-6-phosphate | 0.96 | -0.07 | 0.796 | 0.999 |
| galacturonic acid | 0.96 | -0.07 | 0.721 | 0.999 |
| glucose-6-phosphate | 0.94 | -0.08 | 0.752 | 0.999 |
| glutamic acid | 1.01 | 0.02 | 0.992 | 0.999 |
| glutamine 2TMS minor | 1.03 | 0.04 | 0.895 | 0.999 |
| glutaric acid | 0.86 | -0.22 | 0.683 | 0.999 |
| glycerol-3-galactoside | 0.98 | -0.03 | 0.875 | 0.999 |
| glycocyamine | 1.07 | 0.10 | 0.733 | 0.999 |
| glycolic acid | 1.01 | 0.02 | 0.820 | 0.999 |
| guanidinosuccinate | 0.78 | -0.35 | 0.898 | 0.999 |
| guanosine | 1.01 | 0.02 | 0.698 | 0.999 |
| hexose | 1.04 | 0.05 | 0.866 | 0.999 |
| hexose-6-phosphate | 0.99 | -0.01 | 0.986 | 0.999 |
| hexuronic acid | 1.05 | 0.06 | 0.636 | 0.999 |
| histidine | 1.10 | 0.14 | 0.891 | 0.999 |
| hydroxycarbamate NIST | 1.18 | 0.24 | 0.907 | 0.999 |
| hydroxylamine | 1.30 | 0.38 | 0.834 | 0.999 |
| inosine | 0.99 | -0.02 | 0.987 | 0.999 |
| isohexonic acid | 1.05 | 0.07 | 0.882 | 0.999 |
| isomaltose | 1.06 | 0.08 | 0.856 | 0.999 |
| lactamide | 0.99 | -0.02 | 0.814 | 0.999 |
| lactitol | 1.00 | 0.01 | 0.961 | 0.999 |
| lactulose | 0.80 | -0.31 | 0.869 | 0.999 |
| leucine | 1.04 | 0.05 | 0.715 | 0.999 |
| lyxitol | 1.09 | 0.12 | 0.663 | 0.999 |
| maltotriose | 0.96 | -0.06 | 0.837 | 0.999 |
| mannitol | 1.02 | 0.03 | 0.965 | 0.999 |
| mannonic acid NIST | 1.07 | 0.10 | 0.616 | 0.999 |
| mannose | 1.07 | 0.10 | 0.875 | 0.999 |
| myristic acid | 1.04 | 0.06 | 0.781 | 0.999 |
| N-acetylaspartic acid | 1.32 | 0.40 | 0.960 | 0.999 |
| nicotinamide | 0.96 | -0.06 | 0.944 | 0.999 |
| octadecanol | 1.03 | 0.04 | 0.988 | 0.999 |
| oleamide NIST | 1.21 | 0.28 | 0.742 | 0.999 |
| palmitic acid | 1.00 | -0.01 | 0.880 | 0.999 |
| palmitoleic acid | 0.93 | -0.10 | 0.893 | 0.999 |
| pantothenic acid | 0.97 | -0.04 | 0.974 | 0.999 |
| pentadecanoic acid | 1.10 | 0.14 | 0.871 | 0.999 |
| phosphate | 0.96 | -0.07 | 0.665 | 0.999 |
| pseudo uridine | 1.01 | 0.01 | 0.629 | 0.999 |
| putrescine | 1.02 | 0.03 | 0.973 | 0.999 |
| salicylic acid | 1.26 | 0.33 | 0.948 | 0.999 |
| serine | 1.04 | 0.06 | 0.906 | 0.999 |
| sophorose | 1.03 | 0.05 | 0.840 | 0.999 |
| stearic acid | 0.97 | -0.04 | 0.772 | 0.999 |
| succinate semialdehyde | 1.08 | 0.11 | 0.963 | 0.999 |
| succinic acid | 1.22 | 0.29 | 0.926 | 0.999 |
| UDP-glucuronic acid | 0.95 | -0.08 | 0.734 | 0.999 |
| UDP-N-acetylglucosamine | 1.01 | 0.02 | 0.732 | 0.999 |
| uridine | 0.81 | -0.31 | 0.871 | 0.999 |
| xanthine | 1.02 | 0.03 | 0.812 | 0.999 |
| xylonolactone NIST | 0.97 | -0.04 | 0.833 | 0.999 |

**Table S3.** Difference in abundance of mapped hepatic metabolites between positive control and Selenized yeast group (PC/SY)

| Compound name | Fold Change | log2(FC) | p.value | FDR |
| --- | --- | --- | --- | --- |
| 1-monostearin | 1.99 | 0.99 | 0.015 | 0.523 |
| adenine | 0.76 | -0.40 | 0.023 | 0.523 |
| ascorbic acid | 0.37 | -1.45 | 0.047 | 0.523 |
| beta-glycerolphosphate | 1.86 | 0.90 | 0.040 | 0.523 |
| cholesterol | 1.29 | 0.36 | 0.044 | 0.523 |
| cis-gondoic acid | 1.55 | 0.63 | 0.057 | 0.523 |
| cysteine | 0.53 | -0.91 | 0.024 | 0.523 |
| dodecanol | 1.36 | 0.44 | 0.062 | 0.523 |
| epsilon-caprolactam | 1.45 | 0.54 | 0.056 | 0.523 |
| galacturonic acid | 0.17 | -2.53 | 0.034 | 0.523 |
| gluconic acid | 0.41 | -1.28 | 0.051 | 0.523 |
| glutamine 2TMS minor | 0.23 | -2.14 | 0.056 | 0.523 |
| hydroxylamine | 0.48 | -1.06 | 0.039 | 0.523 |
| inositol-4-monophosphate | 1.95 | 0.96 | 0.061 | 0.523 |
| lactic acid | 0.67 | -0.57 | 0.031 | 0.523 |
| lactulose | 3.07 | 1.62 | 0.011 | 0.523 |
| L-DOPA | 1.41 | 0.50 | 0.056 | 0.523 |
| mannonic acid NIST | 0.21 | -2.27 | 0.055 | 0.523 |
| pentonic acid | 0.45 | -1.14 | 0.008 | 0.523 |
| sophorose | 1.58 | 0.66 | 0.056 | 0.523 |
| spermine | 0.75 | -0.41 | 0.013 | 0.523 |
| succinate semialdehyde | 2.17 | 1.12 | 0.004 | 0.523 |
| uracil | 1.99 | 0.99 | 0.032 | 0.523 |
| uric acid | 1.51 | 0.60 | 0.044 | 0.523 |
| cytidine-5-monophosphate | 1.41 | 0.50 | 0.066 | 0.540 |
| galactonic acid | 0.27 | -1.90 | 0.075 | 0.563 |
| oxamic acid | 1.42 | 0.50 | 0.075 | 0.563 |
| 2-monoolein | 1.36 | 0.44 | 0.085 | 0.609 |
| acetophenone NIST | 0.70 | -0.50 | 0.097 | 0.609 |
| arachidonic acid | 1.50 | 0.58 | 0.087 | 0.609 |
| erythronic acid lactone | 0.74 | -0.43 | 0.098 | 0.609 |
| galactose-6-phosphate | 0.45 | -1.16 | 0.099 | 0.609 |
| hydroxycarbamate NIST | 0.43 | -1.22 | 0.095 | 0.609 |
| oleic acid | 1.22 | 0.29 | 0.103 | 0.612 |
| beta-alanine | 1.18 | 0.24 | 0.112 | 0.616 |
| ethanolamine | 1.77 | 0.82 | 0.108 | 0.616 |
| urea | 2.27 | 1.18 | 0.112 | 0.616 |
| fructose-1-phosphate | 0.66 | -0.59 | 0.119 | 0.637 |
| 2-aminobutyric acid | 2.05 | 1.04 | 0.141 | 0.707 |
| fructose | 1.42 | 0.51 | 0.146 | 0.707 |
| glutaric acid | 1.44 | 0.53 | 0.145 | 0.707 |
| pseudo uridine | 1.50 | 0.59 | 0.136 | 0.707 |
| 1,3,5-trimethylcyanuric acid | 1.18 | 0.24 | 0.315 | 0.729 |
| 2-hydroxyglutaric acid | 1.30 | 0.38 | 0.277 | 0.729 |
| 2-monopalmitin | 1.22 | 0.29 | 0.206 | 0.729 |
| 3,6-anhydro-D-galactose | 1.16 | 0.21 | 0.312 | 0.729 |
| 3-hydroxy-3-methylglutaric acid | 1.56 | 0.64 | 0.351 | 0.729 |
| 3-phosphoglycerate | 0.77 | -0.38 | 0.277 | 0.729 |
| 4-hydroxybutyric acid | 0.89 | -0.17 | 0.389 | 0.729 |
| 65h-benzocphenanthridinone, 11,12-dihydro- NIST | 0.87 | -0.20 | 0.167 | 0.729 |
| alanine | 0.80 | -0.32 | 0.277 | 0.729 |
| alpha-aminoadipic acid | 5.03 | 2.33 | 0.342 | 0.729 |
| aminomalonate | 0.83 | -0.26 | 0.371 | 0.729 |
| arabinose | 1.31 | 0.39 | 0.195 | 0.729 |
| arabitol | 4.22 | 2.08 | 0.204 | 0.729 |
| aspartic acid | 0.71 | -0.50 | 0.338 | 0.729 |
| behenic acid | 1.22 | 0.29 | 0.224 | 0.729 |
| capric acid | 1.15 | 0.21 | 0.382 | 0.729 |
| cerotinic acid | 1.45 | 0.53 | 0.311 | 0.729 |
| citrulline | 1.23 | 0.30 | 0.214 | 0.729 |
| conduritol-beta-epoxide | 0.81 | -0.30 | 0.376 | 0.729 |
| dehydroabietic acid | 1.41 | 0.50 | 0.299 | 0.729 |
| dehydroascorbic acid | 0.28 | -1.83 | 0.169 | 0.729 |
| fructose-6-phosphate | 0.80 | -0.32 | 0.273 | 0.729 |
| glucose-6-phosphate | 0.86 | -0.22 | 0.318 | 0.729 |
| glycine | 1.16 | 0.22 | 0.201 | 0.729 |
| glycolic acid | 0.82 | -0.28 | 0.236 | 0.729 |
| guanine | 1.19 | 0.25 | 0.385 | 0.729 |
| heptadecanoic acid | 0.68 | -0.55 | 0.395 | 0.729 |
| hexitol | 1.34 | 0.43 | 0.218 | 0.729 |
| hexose | 1.30 | 0.38 | 0.317 | 0.729 |
| hexose-6-phosphate | 0.87 | -0.20 | 0.369 | 0.729 |
| hexuronic acid | 0.79 | -0.35 | 0.279 | 0.729 |
| histidine | 0.68 | -0.57 | 0.175 | 0.729 |
| inosine | 1.15 | 0.20 | 0.391 | 0.729 |
| isohexonic acid | 0.68 | -0.56 | 0.175 | 0.729 |
| isoleucine | 1.18 | 0.24 | 0.324 | 0.729 |
| isomaltose | 1.32 | 0.40 | 0.204 | 0.729 |
| lactamide | 1.24 | 0.31 | 0.225 | 0.729 |
| leucine | 1.22 | 0.29 | 0.342 | 0.729 |
| linoleic acid | 1.34 | 0.43 | 0.252 | 0.729 |
| lyxitol | 1.30 | 0.38 | 0.330 | 0.729 |
| myristic acid | 0.87 | -0.20 | 0.285 | 0.729 |
| n-acetyl-d-hexosamine | 1.43 | 0.51 | 0.394 | 0.729 |
| N-carbamoylaspartate | 0.90 | -0.16 | 0.391 | 0.729 |
| nicotinamide | 1.22 | 0.29 | 0.286 | 0.729 |
| oleamide NIST | 0.60 | -0.73 | 0.367 | 0.729 |
| oxoproline | 1.08 | 0.11 | 0.299 | 0.729 |
| palmitic acid | 0.88 | -0.18 | 0.352 | 0.729 |
| pelargonic acid | 0.66 | -0.60 | 0.360 | 0.729 |
| phosphoethanolamine | 1.37 | 0.45 | 0.394 | 0.729 |
| pinitol | 1.45 | 0.53 | 0.167 | 0.729 |
| proline | 1.36 | 0.45 | 0.274 | 0.729 |
| p-tolyl glucuronide | 1.57 | 0.65 | 0.343 | 0.729 |
| putrescine | 1.54 | 0.62 | 0.304 | 0.729 |
| pyrophosphate | 0.88 | -0.19 | 0.314 | 0.729 |
| ribonic acid | 0.62 | -0.69 | 0.368 | 0.729 |
| ribose | 1.24 | 0.30 | 0.380 | 0.729 |
| salicylic acid | 1.44 | 0.53 | 0.355 | 0.729 |
| squalene | 1.54 | 0.62 | 0.220 | 0.729 |
| sucrose | 1.65 | 0.72 | 0.196 | 0.729 |
| tartaric acid | 0.76 | -0.39 | 0.325 | 0.729 |
| threonine | 1.27 | 0.34 | 0.377 | 0.729 |
| tryptophan | 1.12 | 0.17 | 0.374 | 0.729 |
| UDP-glucuronic acid | 0.54 | -0.89 | 0.213 | 0.729 |
| UDP-N-acetylglucosamine | 1.24 | 0.31 | 0.238 | 0.729 |
| valine | 1.35 | 0.43 | 0.243 | 0.729 |
| xanthine | 1.24 | 0.32 | 0.291 | 0.729 |
| xanthosine | 1.19 | 0.25 | 0.270 | 0.729 |
| zymosterol | 0.80 | -0.32 | 0.391 | 0.729 |
| maltotriose | 1.41 | 0.50 | 0.404 | 0.739 |
| malic acid | 0.83 | -0.26 | 0.410 | 0.744 |
| arachidic acid | 1.45 | 0.53 | 0.422 | 0.757 |
| ornithine | 0.88 | -0.18 | 0.427 | 0.760 |
| pantothenic acid | 1.32 | 0.40 | 0.433 | 0.765 |
| glycocyamine | 0.78 | -0.35 | 0.440 | 0.771 |
| docosahexaenoic acid | 1.10 | 0.14 | 0.462 | 0.783 |
| lyxose | 0.87 | -0.20 | 0.458 | 0.783 |
| orotic acid | 1.17 | 0.22 | 0.463 | 0.783 |
| taurine | 5.11 | 2.35 | 0.456 | 0.783 |
| asparagine | 0.83 | -0.27 | 0.470 | 0.784 |
| ketohexose | 0.80 | -0.32 | 0.475 | 0.784 |
| O-phosphoserine | 1.07 | 0.09 | 0.472 | 0.784 |
| ribose-5-phosphate | 0.52 | -0.93 | 0.488 | 0.799 |
| 1-methylinosine NIST | 1.16 | 0.21 | 0.495 | 0.804 |
| lactitol | 1.13 | 0.17 | 0.517 | 0.823 |
| sorbitol | 0.98 | -0.03 | 0.516 | 0.823 |
| tagatose | 1.35 | 0.44 | 0.519 | 0.823 |
| stearic acid | 0.92 | -0.12 | 0.527 | 0.830 |
| methionine sulfoxide | 1.05 | 0.06 | 0.532 | 0.831 |
| glucose | 0.93 | -0.10 | 0.546 | 0.847 |
| beta-sitosterol | 1.09 | 0.12 | 0.564 | 0.848 |
| cellobiose | 1.16 | 0.21 | 0.555 | 0.848 |
| cholesterone | 1.07 | 0.09 | 0.562 | 0.848 |
| N-acetylaspartic acid | 1.30 | 0.38 | 0.557 | 0.848 |
| 5'-deoxy-5'-methylthioadenosine | 1.25 | 0.32 | 0.574 | 0.852 |
| oxalic acid | 0.93 | -0.10 | 0.575 | 0.852 |
| creatinine | 1.65 | 0.73 | 0.614 | 0.878 |
| glucose-1-phosphate | 0.92 | -0.12 | 0.604 | 0.878 |
| palmitoleic acid | 1.17 | 0.23 | 0.613 | 0.878 |
| phenylalanine | 1.08 | 0.11 | 0.602 | 0.878 |
| trans-4-hydroxy-L-proline | 1.13 | 0.17 | 0.607 | 0.878 |
| chlorogenic acid | 1.37 | 0.46 | 0.626 | 0.883 |
| lanosterol | 1.14 | 0.19 | 0.622 | 0.883 |
| threonic acid | 0.96 | -0.05 | 0.641 | 0.897 |
| 1-monoolein | 1.31 | 0.39 | 0.654 | 0.909 |
| 1,2-anhydro-myo-inositol NIST | 0.95 | -0.07 | 0.667 | 0.909 |
| glycerol-alpha-phosphate | 1.04 | 0.06 | 0.672 | 0.909 |
| guanidinosuccinate | 3.02 | 1.59 | 0.670 | 0.909 |
| hypoxanthine | 1.14 | 0.19 | 0.664 | 0.909 |
| galactose | 0.97 | -0.04 | 0.679 | 0.913 |
| 2,5-dihydroxypyrazine NIST | 1.12 | 0.17 | 0.699 | 0.933 |
| butyrolactam NIST | 0.94 | -0.09 | 0.703 | 0.933 |
| glyceric acid | 1.28 | 0.36 | 0.711 | 0.938 |
| tocopherol alpha- | 0.99 | -0.02 | 0.719 | 0.942 |
| adenosine | 0.98 | -0.04 | 0.733 | 0.946 |
| guanosine | 0.96 | -0.05 | 0.736 | 0.946 |
| serine | 1.07 | 0.10 | 0.730 | 0.946 |
| methionine | 1.12 | 0.17 | 0.742 | 0.948 |
| 1-monoheptadecanoyl glyceride NIST | 1.05 | 0.08 | 0.771 | 0.952 |
| citric acid | 1.09 | 0.12 | 0.764 | 0.952 |
| cystine | 1.04 | 0.06 | 0.787 | 0.952 |
| fumaric acid | 0.94 | -0.10 | 0.784 | 0.952 |
| isothreonic acid | 1.12 | 0.17 | 0.778 | 0.952 |
| myo-inositol | 1.06 | 0.08 | 0.790 | 0.952 |
| pentadecanoic acid | 1.01 | 0.01 | 0.789 | 0.952 |
| phosphate | 1.14 | 0.19 | 0.793 | 0.952 |
| pyrrole-2-carboxylic acid | 1.07 | 0.10 | 0.783 | 0.952 |
| uridine | 0.66 | -0.61 | 0.782 | 0.952 |
| 2-hydroxypyrazinyl-2-propenoic acid ethyl ester NIST | 0.98 | -0.03 | 0.813 | 0.954 |
| 5-aminovaleric acid | 1.08 | 0.10 | 0.818 | 0.954 |
| adenosine-5-monophosphate | 0.92 | -0.13 | 0.807 | 0.954 |
| lysine | 1.08 | 0.12 | 0.812 | 0.954 |
| maleimide | 0.96 | -0.06 | 0.815 | 0.954 |
| 4-aminobutyric acid | 1.03 | 0.04 | 0.868 | 0.971 |
| 5-methoxytryptamine | 0.87 | -0.21 | 0.889 | 0.971 |
| enolpyruvate NIST | 0.97 | -0.04 | 0.872 | 0.971 |
| glycerol | 1.24 | 0.31 | 0.844 | 0.971 |
| glycerol-3-galactoside | 1.00 | 0.00 | 0.847 | 0.971 |
| hydroquinone | 0.97 | -0.05 | 0.885 | 0.971 |
| levoglucosan | 0.98 | -0.03 | 0.882 | 0.971 |
| propane-1,3-diol NIST | 1.05 | 0.07 | 0.879 | 0.971 |
| ribulose-5-phosphate | 1.16 | 0.21 | 0.853 | 0.971 |
| succinic acid | 1.16 | 0.22 | 0.877 | 0.971 |
| xylitol | 0.96 | -0.07 | 0.857 | 0.971 |
| xylose | 1.00 | 0.00 | 0.847 | 0.971 |
| glutamic acid | 1.01 | 0.02 | 0.900 | 0.977 |
| D-erythro-sphingosine | 1.05 | 0.08 | 0.920 | 0.983 |
| fucose | 1.19 | 0.25 | 0.920 | 0.983 |
| methanolphosphate | 1.13 | 0.17 | 0.912 | 0.983 |
| ethanol phosphate NIST | 1.10 | 0.13 | 0.934 | 0.987 |
| pyruvic acid | 0.85 | -0.24 | 0.934 | 0.987 |
| 1,2,4-benzenetriol | 1.02 | 0.03 | 0.993 | 0.993 |
| 1-hexadecanol | 1.01 | 0.01 | 0.951 | 0.993 |
| 3-aminoisobutyric acid | 1.00 | 0.00 | 0.967 | 0.993 |
| benzoic acid | 1.03 | 0.04 | 0.992 | 0.993 |
| galactinol | 0.85 | -0.24 | 0.989 | 0.993 |
| mannitol | 1.04 | 0.06 | 0.990 | 0.993 |
| mannose | 1.09 | 0.12 | 0.960 | 0.993 |
| octadecanol | 1.07 | 0.10 | 0.979 | 0.993 |
| thymidine | 1.10 | 0.14 | 0.952 | 0.993 |
| tyrosine | 1.00 | -0.01 | 0.980 | 0.993 |
| xylonolactone NIST | 0.99 | -0.01 | 0.979 | 0.993 |
