# Supplementary figures and images for: Organic selenium supplement partially alleviated diquat-induced oxidative insults and hepatic metabolic stress in nursery pigs
Source: Br J Nutr. 2020 Mar 2;124(1):23–33. doi: 10.1017/S0007114520000689 (PMC7512145; doi:10.1017/S0007114520000689)

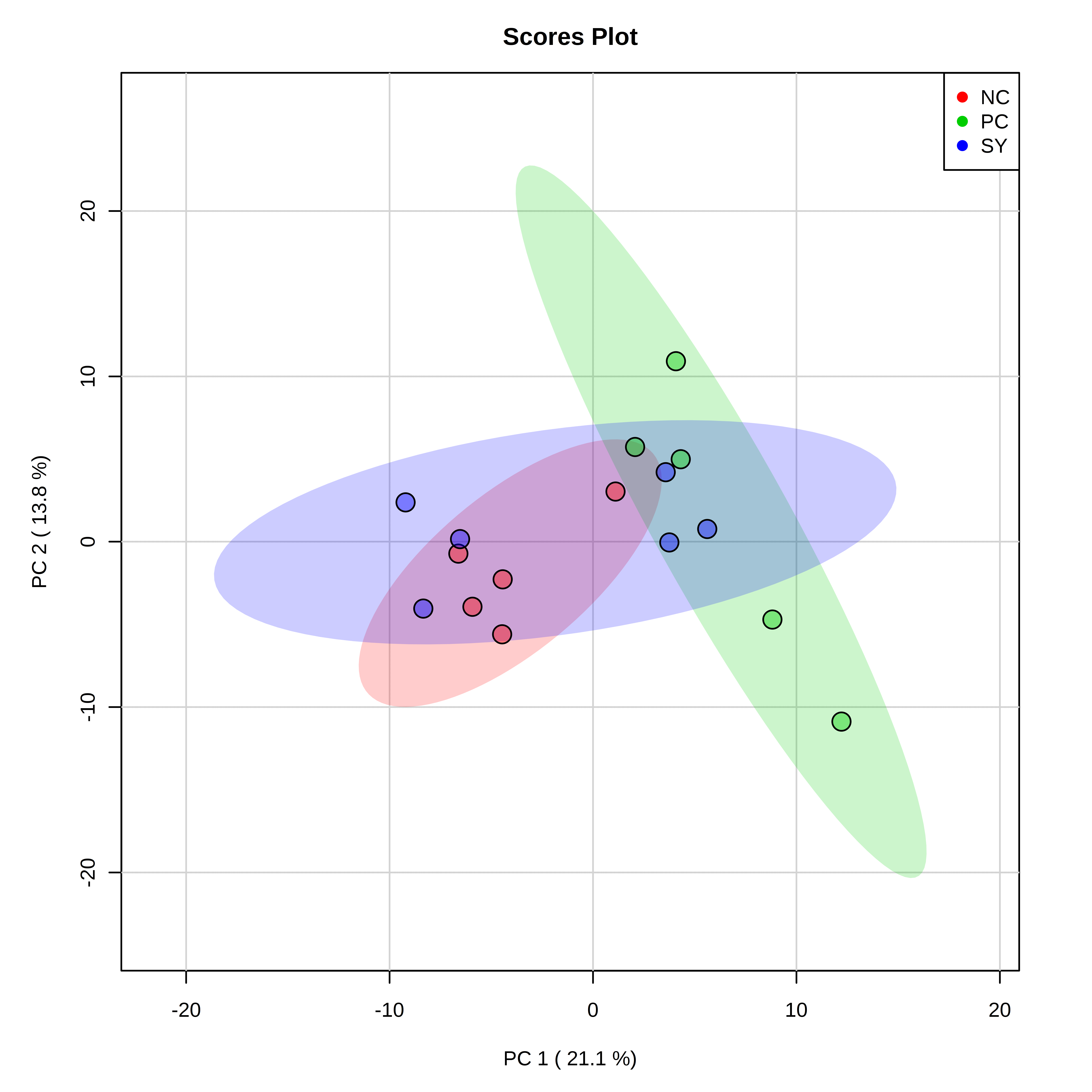

Supplement: Supplementary file 1 [file S0007114520000689sup.zip › S0007114520000689sup001.tiff]
